# Automated vs. manual case investigation and contact tracing for pandemic surveillance: Evidence from a stepped wedge cluster randomized trial

**DOI:** 10.1016/j.eclinm.2022.101726

**Published:** 2022-11-12

**Authors:** Cameron Raymond, Derek Ouyang, Alexis D'Agostino, Sarah L. Rudman, Daniel E. Ho

**Affiliations:** a Regulation, Evaluation, and Governance Lab, Stanford University, Stanford, CA, USA; b County of Santa Clara Public Health Department, San Jose, CA, USA

**Keywords:** COVID-19, Contact tracing, Health disparities

## Abstract

**Background:**

Case investigation and contact tracing (CICT) is an important tool for communicable disease control, both to proactively interrupt chains of transmission and to collect information for situational awareness. We run the first randomized trial of COVID-19 CICT to investigate the utility of manual (*i.e.*, call-based) vs. automated (*i.e.*, survey-based) CICT for pandemic surveillance.

**Methods:**

Between December 15, 2021 and February 5, 2022, a stepped wedge cluster randomized trial was run in which Santa Clara County ZIP Codes progressively transitioned from manual to automated CICT. Eleven individual-level data fields on demographics and disease dynamics were observed for non-response. The data contains 106,522 positive cases across 29 ZIP Codes.

**Findings:**

Automated CICT reduced overall collected information by 29 percentage points (SE = 0.08, *p* < 0.01), as well as the response rate for individual fields. However, we find no evidence of differences in information loss by race or ethnicity.

**Interpretations:**

Automated CICT can serve as a useful alternative to manual CICT, with no substantial evidence of skewing data along racial or ethnic lines, but manual CICT improves completeness of key data for monitoring epidemiologic patterns.

**Funding:**

This research was supported in part by the Stanford Office of Community Engagement and the Stanford Institute for Human-Centered Artificial Intelligence.

**Research in context**

**Evidence before this study**

Case investigation and contact tracing (CICT) is an important tool for communicable disease control and pandemic awareness. Previous work has argued that access to complete and representative data is critical for an effective and equitable response, but no randomized trials exist to assess different forms of contact tracing.

**Added value of this study**

We report on the first randomized controlled trial of contact tracing in operation. In a large jurisdiction, we demonstrate that automated (survey-based) contact tracing results in substantial information loss, but that such information loss does not appear to be magnified across racial and ethnic subgroups.

**Implications of all the available evidence**

Accurate, representative, and complete public health data is crucial to pandemic response. Automated CICT can serve as a useful alternative, albeit with considerable informational loss, to manual CICT. These findings inform how to effectively use contact tracing, especially as many jurisdictions are searching for alternatives to resource-intensive manual contact tracing.

## Introduction

Health departments rely on large-scale, time-sensitive streams of disease data for situational awareness and decision making in pandemic response. Case investigation and contact tracing (CICT) is used to identify and suppress disease outbreaks, but also plays a critical role in collecting public health information. These data are needed for understanding disease dynamics and targeting public health interventions (*e.g.*, the role of travel or gatherings in disease transmission),^1^, ^2^, ^3^, ^4^ as well as collecting demographic information that is central to understanding disease disparities.^1^^,^^5^, ^6^, ^7^, ^8^

Throughout the COVID-19 pandemic, time-intensive manual CICT, wherein contact tracers interview patients by phone, has been the default for most of the U.S. Given the resources required, a persistent challenge has been balancing human involvement and technological automation in CICT. A second form of CICT are mobile phone applications, which automatically record close contacts via bluetooth. Previous work has argued that such applications require adoption rates of 50% to control outbreaks.^9^ While this approach has been utilized to varying effect in much of the world, population uptake, privacy, and security have been identified as barriers to its effectiveness in the U.S.^10^ Other approaches using electronic health records have been attempted at the local level.^11^

Manual CICT, meanwhile, has been costly. The state of California spent over $2.2 billion on CICT, much of which went to call-based efforts.^12^ Massachusetts spent nearly $160 million on CICT, but abandoned efforts in 2021.^13^ Manual CICT is also difficult to scale: a study of CICT in the US found that 40% of individuals with COVID-19 were not reached for an interview.^14^ Due to the length and magnitude of the pandemic, call-based efforts have proven unsustainable. As a result, balancing the cost and effectiveness of different forms of CICT is of increasing practical and scholarly importance.^15^

The objective of this paper is to assess the benefits and costs of a third approach: automated surveys. This alternative informs patients about isolation and collects critical health information, much in the same way that manual CICT does, but through an online questionnaire. Automated surveys also benefit from being cost-effective and unreliant on widespread uptake of application-based contact tracing software. Human involvement can, in turn, be focused on extracting data insights or following up with high priority patient needs. Thus, automated surveys have the potential to address the concerns of both manual and app-based CICT. Yet automated CICT may also limit and skew the information health departments receive. Automated CICT may reduce the willingness of patients to offer potentially sensitive information. Differential non-response across demographic groups could also mask health inequalities.

We evaluate automated CICT in an actual public health operation in Santa Clara County (SCC), home to 1.92 million residents. SCC, along with other Bay Area counties, instituted the first shelter-in-place order in the U.S.^16^ We utilize a novel stepped wedge cluster randomized trial where ZIP Codes transition from manual (call-based) to automated (survey-based) CICT over the course of 3 months. This design met several goals. First, the SCC Public Health Department had already planned for a transition to automated CICT, but this design enabled rigorous evaluation of its impacts. Second, in support of local health equity objectives, we were able to retain the potential benefits of manual CICT in the most vulnerable areas for the longest period.

Our study contrasts the existing evidence base for CICT, which has relied on observational designs or simulations. In those designs, confounding variables or strong modeling assumptions can limit an understanding of the causal impact of CICT.^10^^,^^17^, ^18^, ^19^, ^20^ To our knowledge, this is the first randomized study of COVID-19 contact tracing in operation.^10^^,^^17^^,^^18^

Our results show that ZIP Codes randomized out of manual CICT experienced significant information loss, defined as a drop in the proportion of key fields regarding demographics and disease dynamics that were meaningfully answered. However, we do not find evidence of differential effects across racial or ethnic lines, indicating that automated CICT did not further mask racial or ethnic disparities in health outcomes. These results demonstrate that automated CICT is viable – and preferable to no CICT whatsoever – when manual CICT becomes too resource intensive.

Our paper proceeds as follows. In Methods, we discuss the intervention that replaced manual with automated CICT, describe data sources, and detail the design of the stepped wedge cluster randomized trial, as well as methods for systematically measuring race and ethnicity. In Results, we verify that the design achieved covariate balance, provide results on the causal effects of automated CICT on the completeness of critical public health data, and test for differential effects by race and ethnicity. In Discussion, we discuss the implications and limitations of this work.

## Methods

### Intervention

Positive cases in this study receive one of two interventions. The first is *Manual CICT* (call-based or control), which involves a telephone call from a trained contact tracer who gathers information surrounding the case. Depending on capacity, contact tracers will attempt to call a participant up to six times. The second is *Automated CICT* (survey-based or treatment), which is sent via text message/email. Surveys are answered on a web browser and consist of free-text, multiple choice, and drop down inputs.^21^

Both conditions have similar questions, as the survey was drafted to emulate manual CICT. We focused on eleven questions deemed especially important by the SCC Public Health Department, spanning demographics (race/ethnicity, gender, sexual orientation, language), and disease dynamics (signs/symptoms, employer, travel history, gathering history, ability to isolate, contacts generated, congregate settings). Table 1 provides sample questions (for the full list, see Appendix A). We note that there are some small but negligible wording differences. We focus on the overall effect across questions but provide question-specific results in Appendix E.

**Table 1:** Sample of questions from call-based vs. automated survey script for CICT.

		Manual CICT (Phone Call)	Automated CICT (Online Survey)
Question	Introduction	I am with the Santa Clara County Department of Public Health calling in regards to your COVID-19 test result. First let me say that everything you and I talk about is confidential. I need to let you know your COVID-19 test result has come back positive. This means you do have coronavirus disease or COVID-19. We are calling everyone who has tested positive in the state to share information about how to keep themselves and their families safe and to collect information so that we can prevent further spread of the virus. I also want to check on how you are doing, see if you need any support right now, and answer any COVID-19 questions you may have.	Hi, this is your Santa Clara County Department of Public Health reaching out about an important health issue. We are reaching out to you because you have tested positive for COVID-19. This virus is very contagious, so it is important to keep it from spreading to others. The Santa Clara County Department of Public Health is working hard to slow the spread of COVID-19. You can help us by answering a few very important questions. The answers you give will help us protect you, the people in your house, and your community.
	Symptoms	How are you feeling? Are you currently experiencing any symptoms? Some of the symptoms of COVID-19 are well known, while others are a little hard to recognize. So I'd like to read through a list of symptoms — could you tell me if you have experienced any of these symptoms?	Have you experienced any of these COVID-related symptoms recently? Click the arrow to view a list of COVID-related symptoms. At the end of the survey, we will provide you with information about COVID-19 symptoms and resources.
	Gathering History	In the 7 days before your symptoms started, did you do anything or go anywhere where you were around 4 or more people not living in your household?	In the 7 days before your symptoms started/or receiving your positive test, did you attend any large gatherings?
	Ability to Isolate	What concerns you about being able to self-isolate? How sure are you that you are able to safely isolate at home? How safe do you feel in your home?	Are you able to safely self-isolate at home, away from others? This information can help us understand your current status at home and we may be able to help you.

CICT = Case investigation and contact tracing.

### Data and outcomes

All positive cases within 29 eligible ZIP Codes during the study period were included for analysis. We observe 106,522 positive cases across the 29 eligible ZIP Codes. Individual-level data is entered in the state-managed system used to record and investigate COVID-19 cases in California (CalCONNECT). Thus, our study has a unique insight into data actually used for pandemic surveillance. Responses from manual CICT are entered directly by the contact tracer, while responses from automated CICT are transferred programmatically to CalCONNECT.

Our outcome of interest is the proportion of the eleven data fields that were answered by participants. Each is observed for non-response, including “unknown”, “declined to answer”, and null. While CICT is one of the main ways in which contextual information is collected, other sources, like COVID-19 laboratory test questionnaires, also ask some similar questions. Thus, even if someone does not complete either form of CICT, some information may be known (see Appendix F).

Our estimand of interest is information loss, defined as the change in the proportion of fields filled out. While our primary outcome is the overall completion rate, we also provide question-level estimates in Appendix E.

Initially, this study was designed to understand the effect of CICT on community transmission (*i.e.*, case rates in a given ZIP Code per 100k). While this was the prespecified primary hypothesis, non-compliance as a result of the Omicron surge meant we did not achieve sufficient power to analyze this outcome (see Appendix I). As such, we focus on information loss.

While race/ethnicity data is similarly evaluated for non-response, it is also transformed to six categories of Hispanic, Asian American and Pacific Islander (AAPI), White, Black, American Indian and Alaskan Native (AIAN), or Other Race. Due to potentially endogenous non-response, race and ethnicity may not be observed for each participant. We hence also construct an exogenous measure of race by imputing it for each participant using Bayesian Improved Firstname Surname Geocoding (BIFSG).^22^^,^^23^ Appendix D provides details on the procedure. Gender is transformed to male, female, non-binary, or other. Language is reduced to English, Spanish, or some other language.

### Randomization and treatment assignment

We designed a stepped wedge (*i.e.*, randomized rollout) cluster randomized trial to rigorously assess the effects of transitioning from manual to automated CICT. We cluster our randomization at the ZIP Code level. Between December 15, 2021 and February 5, 2022, we randomized the “step down” date of eligible ZIP Codes at which point they transitioned from manual to automated CICT. Before a ZIP Code steps down, participants are assigned a contact tracer who attempts manual CICT. If the call is not answered, participants are also sent the automated survey, but because a call attempt was initiated, such participants are still considered “assigned” to manual CICT. After a ZIP Code steps down, participants are no longer routed to manual CICT, and instead only the automated survey is sent.

**Fig. 1:**
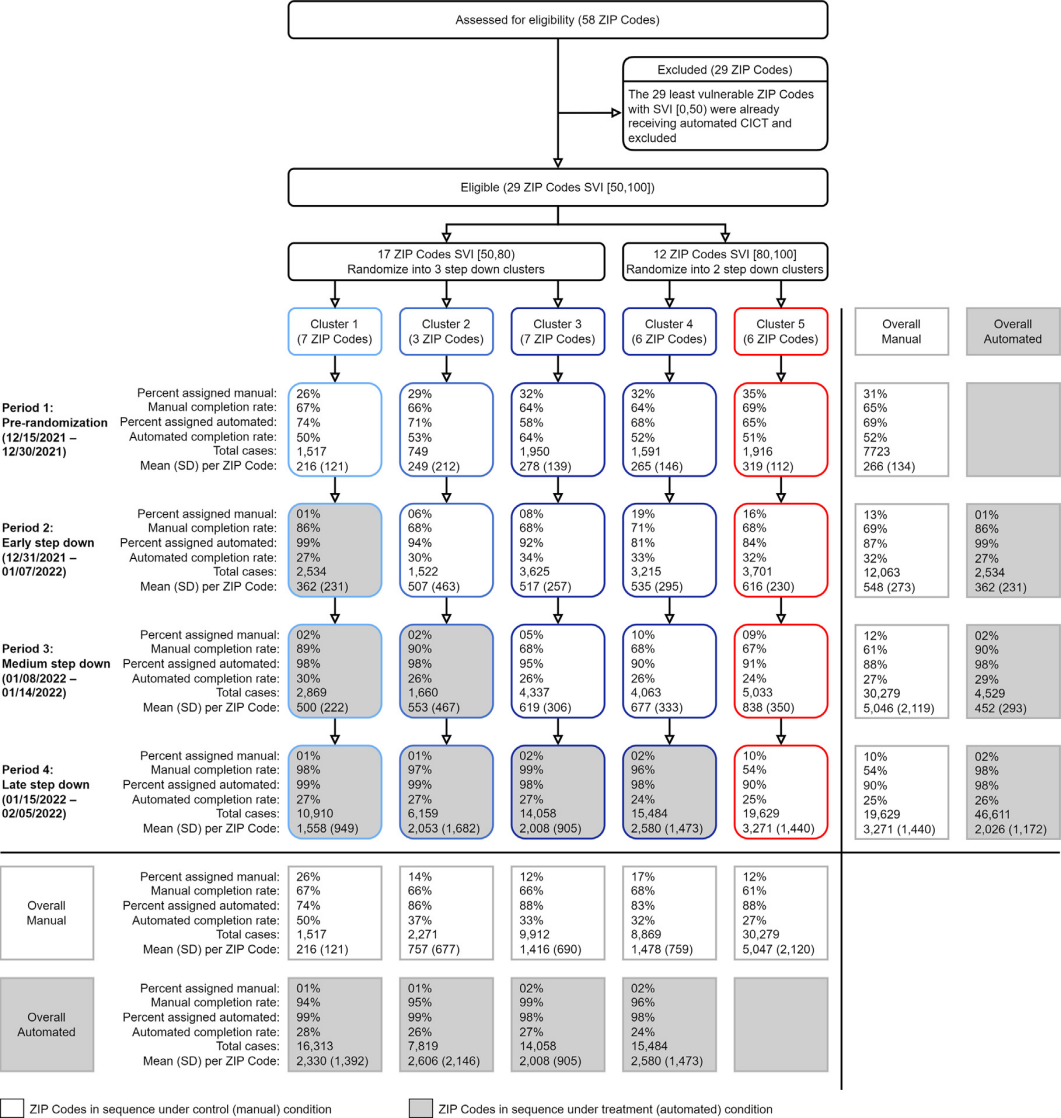
CONSORT SW-CRT Flowchart for the stepped wedge cluster randomized trial. This flowchart illustrates how 29 of the 58 ZIP Codes in Santa Clara County are randomized into 5 step down clusters. The 29 eligible ZIP Codes are segmented by vulnerability. Then the less vulnerable ZIP Codes are randomized into 3 step down clusters, corresponding to the time that they transition from attempting manual CICT to the automated, survey-based approach to CICT. The more vulnerable ZIP Codes are randomized into two clusters, one which steps down in the late step down period, and one that remains in the manual CICT branch. Manual and automated completion rate is the proportion of participants who completed the treatment they were assigned to (*i.e.*, answered the phone call or completed the automated survey). CICT = Case investigation and contact tracing; SVI = Social Vulnerability Index; SD = Standard deviation.

Step down dates are stratified by the Centers for Disease Control and Prevention Social Vulnerability Index (SVI) to retain call-based CICT for the highest vulnerability areas for the longest duration.^24^^,^^25^ The 29 lowest SVI ZIP Codes had already been withdrawn from manual CICT and were ineligible for randomization. The remaining 29 eligible ZIP Codes were segmented into two SVI strata, SVI [50,80) and SVI [80,100], and allocated to one of four step down sequences. ZIP Codes with SVI [50,80) were randomized into early, medium, and late step down clusters. ZIP Codes with SVI [80,100] were randomized into a late step down cluster and a never step down cluster. The full stepped wedge design can be seen in Fig. 1, which outlines this protocol, the size of each step down cluster, and descriptive statistics at each stage in the study. Randomization was done by the researchers with a random number generator prior to the study period. The allocation sequence was made available to SCC Public Health leadership, but not to those performing manual CICT or study participants.

In addition to randomizing the step down date for each ZIP Code, we also randomized the order in which participants were assigned to manual CICT. This was necessary as capacity constraints meant that not every participant in a control (manual) ZIP Code could be called, especially during the Omicron surge which overwhelmed CICT capabilities from December 20, 2021 (See Appendix C for an overview of CICT coverage during the study period). 87% of overall cases in the control condition were *not* assigned to manual CICT. Given the ease with which our treatment can be deployed, automated CICT was used in these instances. We refer to these as “overflow” cases and detail how we model them as a form of non-compliance in Section 4.3.

Another constraint for our randomization was with respect to the treatment of household units. If someone in a household was assigned manual CICT, then all subsequent cases in that household were also assigned manual CICT, even if that ZIP Code had since stepped down. This was done by the SCC Public Health Department to maintain consistency in the treatment within households and only forms a small proportion (1.6%) of cases.

### Sample size

The timeframe for this study corresponded with the SCC Public Health Department's plan to transition away from manual CICT. As such, our power analysis informed the outcomes suitable for study. Monte-Carlo simulations using historical data were run prior to the study period to determine the minimum detectable effect size for our outcomes of interest. We simulated the randomization protocol outlined in Section 4.1, as well as the two-way fixed effects estimator described in Section 4.3. The intracluster correlation coefficient for completion rate among participants within a ZIP Code prior to the study period was 0.04. Given 100k cases, our study had 80% power to detect 12 percentage point changes in information loss assuming manual CICT coverage of 10% and a significance level of 0.05. This is conservative relative to per-protocol completion rates prior to randomization.

Low manual CICT coverage reduces the number of control cases available and statistical power. As previously noted, clustering our treatment assignment by geography was initially done to understand the effect of CICT on community transmission (*i.e.*, case rates in a given ZIP Code per 100k). The number of overflow cases, however, meant that we would only be able to detect unreasonably large effect sizes (*e.g.*, a +70% increase in case rates at 20% manual CICT coverage). Health outcomes are also at a greater risk for within-cluster contamination relative to informational outcomes.^25^ As such, we focus our attention on informational outcomes.

### Statistical analysis

To formally assess the causal effect of automated CICT on information loss (*i.e.*, the completion of eleven data fields), we adopt a difference-in-differences approach.^26^ This compares the post-step down cluster to those in that same cluster pre-step down, as well as those in control ZIP Codes. By taking into account temporal trends and existing differences in ZIP Code baseline rates, we can estimate the effect of CICT on an intention-to-treat (ITT) basis. We analyze the data at the individual level as a repeated-cross section.

The leverage for causal inference in stepped wedge designs is similar to that of difference-in-differences analysis in observational settings. One is required to make an assumption of parallel trends: that is, while treatment and control groups may have differing base rates in the outcome, their trends in outcomes should be the same. Randomization should make this assumption trivial; however, our stratified randomization schema means that we must assume that the [80,100] SVI stratum has parallel trends as the [50,80) SVI stratum. This appears to be the case. However, our results are also robust to analyzing the data by SVI strata separately, in which case the parallel trends assumption is trivial due to our randomization, yielding similar estimates (see Appendix J). Thus, for the sake of clarity, here we analyze the data in its totality.

We estimate the ITT effect using a two-way fixed effects (TWFE) linear regression with robust standard errors clustered at the ZIP Code level to account for our clustered treatment assignment and heteroscedasticity. In addition to week and ZIP Code fixed effects, we also control for SVI stratum, taking into account our randomization protocol.

As discussed earlier, there are a large number of “overflow” cases which were randomized into manual CICT but were instead assigned automated CICT due to capacity constraints. To account for this form of non-compliance, we also estimate the local average treatment effect (LATE) adopting an instrumental variable (IV) approach.^27^ To contrast the two estimands, the ITT effect is the effect of the intervention as-randomized, but not accounting for noncompliance. The LATE can be thought of as the effect of the intervention on the subgroup of cases whose CICT protocol was affected by randomization. The LATE is often the more policy-relevant estimand since, in principle, one could always allocate more resources to increasing manual CICT call coverage. Thus, while we include ITT estimates according to our study protocol, we primarily report the LATE estimates.

We use our randomization protocol as an instrument for the treatment assigned, using the common two-stage least squares (2SLS) estimator.^28^ By definition this is exogenous, and the first-stage F-statistic being 693 (*p* < 0.01) confirms that the ZIP Code randomization is a strong instrument. Other work has suggested dropping non-compliers in stepped wedge trials to identify the LATE, which provides similar results.^29^ Since our data is in the form of an individual-level repeated cross section, we opt to model non-compliance through the IV analysis and report the 2SLS estimates. As with our ITT analysis, we include week, ZIP Code, and SVI stratum fixed effects and cluster the standard errors at the ZIP Code level.

### Ethics statement

This study examined COVID-19 case investigation and contact tracing data from existing databases used by public health jurisdictions. The Santa Clara County Public Health Department and Stanford University, Stanford, California, deemed the work public health surveillance; the Revised Common Rule deems “public health surveillance activities” not subject to IRB oversight under 45 CFR § 46. Thus, it was not submitted for IRB approval, but was subjected to privacy and compliance review by Santa Clara County.

### Role of funding source

No study sponsors were involved in study design; in the collection, analysis, and interpretation of data; in the writing of the report; or in the decision to submit the paper for publication. All authors had access to and verified all the data in the study. D.E.H. was responsible for the decision to submit the manuscript.

## Results

### Covariate balance

To verify randomization, Table 2 presents covariate balance statistics for treatment compared to eligible control ZIP Codes at each period of the study. This is done on an ITT basis, meaning that cases are grouped by the randomization protocol, not the treatment with which they were ultimately assigned. We observe some imbalances for the race/ethnicity feature due to the small number of AIAN participants, which we attribute to sampling variation. Ultimately, we note no systematic imbalances that could threaten the validity of our study.

**Table 2:** Balance statistics for positive cases in treatment ZIP Codes compared to eligible control ZIP Codes in the three step down clusters.

	Overall	SVI [50,80)						SVI [80,100]		
		Early step down period			Medium step down period			Late step down period		
		Treatment	Control	*p*	Treatment	Control	*p*	Treatment	Control	*p*
Age	31.92 (19.34)	32.75 (19.04)	32.50 (18.91)	0.78	31.88 (19.20)	31.27 (18.69)	0.47	31.74 (19.77)	31.15 (19.70)	0.33
Race/Ethnicity										
Hispanic	42.4%	26.3%	34.1%	0.36	24.3%	32.8%	0.06	53.7%	54.9%	0.03
AAPI	25.1%	35.0%	18.8%		37.0%	22.9%		23.6%	16.9%	
White	14.7%	19.6%	25.3%		14.9%	20.0%		7.1%	11.9%	
Black	2.1%	2.3%	3.3%		2.4%	2.3%		1.4%	2.1%	
AIAN	0.4%	0.2%	0.3%		0.3%	0.1%		0.3%	0.5%	
Other Race	15.3%	16.6%	18.3%		21.1%	21.9%		13.9%	13.7%	
Gender										
Female	51.9%	52.7%	53.2%	0.72	52.0%	50.8%	0.22	51.8%	51.6%	0.48
Male	48.0%	47.3%	46.8%		48.0%	49.0%		48.2%	48.3%	
Non-Binary	0.1%	0.0%	0.0%		0.1%	0.1%		0.0%	0.1%	
Other	0.1%	0.0%	0.0%		0.0%	0.1%		0.0%	0.0%	
Language										
English	73.0%	87.9%	86.9%	0.48	82.7%	76.0%	0.03	62.0%	61.0%	0.53
Spanish	22.0%	7.3%	9.8%		12.1%	20.0%		31.0%	34.0%	
Other	5.0%	4.7%	3.3%		5.3%	4.0%		7.0%	5.0%	
Total Cases	106,522	2,534	5,147		4,529	4,337		15,484	19,629	
Total ZIP Codes	29	7	10		10	7		6	6	
Date Range	12/18/21–02/05/22	12/31/21–01/07/22			01/08/22–01/14/22			01/15/22–02/05/22		

**Fig. 2:**
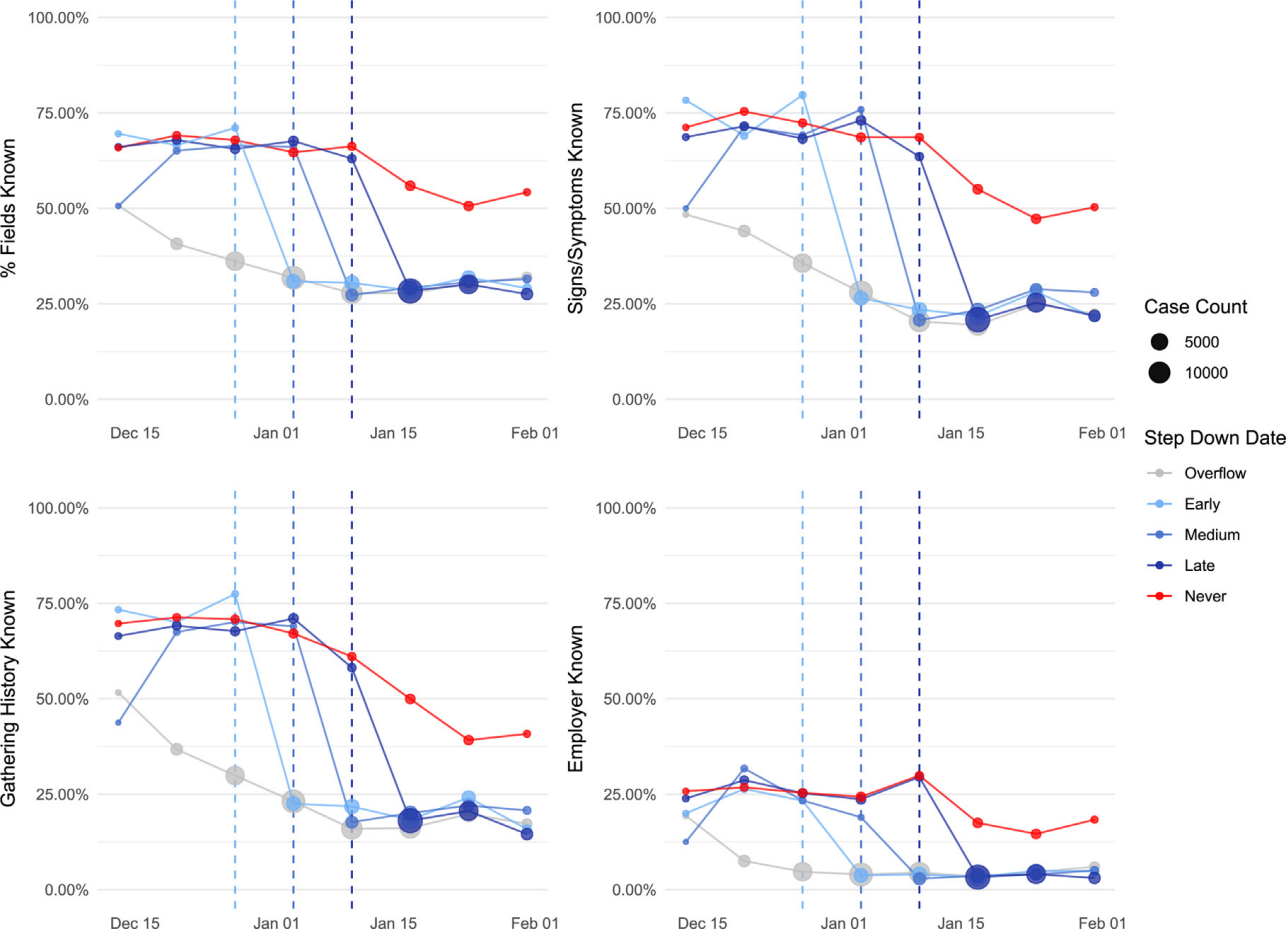
Step down cluster-by-week panel of response rate over time for overall response rate (top left) as well as key individual fields such as symptoms (top right), recent gathering history (bottom left), and employer (bottom right). Time series are colored according to the date at which that cluster of ZIP Codes randomly transitioned from manual to automated CICT, corresponding with the vertical dashed lines. The “Overflow” time series represents all cases randomized out of manual CICT due to capacity constraints. Point size corresponds to the number of cases for that cluster on that week. CICT = Case investigation and contact tracing.

These correspond with the three vertical dashed lines in Fig. 2. ZIP Codes with SVI [50,80) were eligible for being stepped down on either Dec. 31, 2021 or Jan. 8, 2022, with the remaining [50,80) SVI ZIP Codes being stepped down on Jan. 15, 2022. ZIP Codes with SVI [80,100] were either stepped down on Jan. 15, 2022 or retained as the “never” category. Note that cases are clustered by our intention-to-treat (ITT) based on our randomization protocol. Balance is calculated within the step down period date range and SVI strata. Mean age is reported along with standard deviation in parentheses. Balance is assessed at the individual level by regressing the treatment assignment on the balance variable with robust standard errors clustered at the ZIP Code level. p values are calculated via an F-test of overall significance which tests whether including the balance variable in the regression increases predictive power relative to an intercept-only model, allowing for one p value per variable. Failing to reject this test indicates that the balance variable is not meaningfully related to the treatment assignment.

AAPI = Asian American and Pacific Islander; AIAN = American Indian and Alaska Native; SVI = Social Vulnerability Index.

### Information loss

Fig. 2 plots the information loss from transitioning to automated CICT. The top left panel depicts per-case completeness of eleven critical data fields. Each time series depicts cases from ZIP Codes randomly selected to transition on that date. With each cluster, we observe a sharp reduction in information corresponding precisely to the transition from manual to automated CICT. Following the transition, the percentage of fields known per-case is cut in half. The other panels plot similar information loss on three particular fields of high public health relevance, namely symptoms, gathering history, and employer information (full results in Appendix B).

As seen in Table 3, transitioning to automated CICT caused a statistically significant reduction in overall information of 29 percentage points (pp) (SE = 0.08, *p* < 0.01). We find an insignificant 19pp reduction (SE = 0.13, *p* = 0.13) in gathering history completeness. Notably, we estimate a significant 40pp reduction (SE = 0.13, *p* < 0.01) in the rate at which symptoms are known and a significant 32pp reduction (SE = 0.08, *p* < 0.01) in the rate at which a case's employer is known. Appendices F–H provide robustness checks in the form of observational analyses, sensitivity of our results to different model specifications, and a per-protocol analysis, respectively, all of which further confirm our findings.

**Table 3:** The effect of automated CICT on per-case completeness spanning eleven critical data fields.

	Dependent variable:	
	% Fields known	
	ITT Estimates	IV Estimates
	(1)	(2)
Randomized to Automated	−0.02∗∗∗ (0.01)	
Assigned to Automated		−0.29∗ (0.08)
High SVI	0.00 (0.00)	−0.003∗ (0.00)
Observations	106,522	106,522
Adjusted R^2^	0.04	0.13
Residual Std. Error (DF = 106,485)	0.27	0.24

∗*p* < 0.01.

Outcome variable is the percentage of fields filled out. Regression (1) presents the ITT (as randomized) effect, via two-way fixed effects linear regression. Regression (2) presents the LATE which also uses a two-way fixed effects linear regression, but uses the ZIP Code randomization as an instrument for the treatment which is ultimately assigned. This accounts for noncompliance, primarily in the form of overflow cases due to capacity constraints. Both regressions also control for SVI strata and have standard errors clustered at the ZIP Code level in accordance with our randomization protocol. Standard errors are presented in parentheses.

CICT = Case investigation and contact tracing; DF = Degrees of freedom; ITT = Intention-to-treat; IV = Instrumental variables; LATE = Local average treatment effect; SVI = Social Vulnerability Index.

### Differential effects by race

Our race/ethnicity imputation method allows for an investigation into differential effects of automated CICT by race/ethnicity. BIFSG predictions are completely exogenous to the treatment administered. Our subgroup analysis first estimates the LATE for each BIFSG category. We then test the difference in LATE estimates for each BIFSG category with a one-way ANOVA. Due to insufficient sample sizes, we exclude those imputed as AIAN (n = 65) and Other Race (n = 424) in our subgroup analysis.

We find no evidence of differential effects of contact tracing on overall information loss by race/ethnicity among Hispanic (LATE = −0.30, SE = 0.06, *p* < 0.01), White (LATE = −0.31, SE = 0.20, *p* = 0.11), AAPI (LATE = −0.26, SE = 0.13, *p* = 0.04), and Black (LATE = −0.32, SE = 0.51, *p* = 0.53) imputed cases (F(3,106029) = 0.04, *p* = 0.99). See Appendix E for a comparison of the overall estimates and the subgroup estimates for total information loss, as well as individual fields. While only Hispanic and AAPI participants saw estimates that met statistical significance (*i.e.*, *p* < 0.05), the point estimates across races were very similar. Thus even while other racial and ethnic categories are not statistically significant, the effect of automated CICT does not appear statistically distinguishable across race.

Similarly, we find little evidence for differential effects for any individual data field, when using self-reported race and only imputing missing observations, or when using a probabilistic weighted estimator (see Appendix E). Thus, despite a reduction in data completeness in automated CICT, we find no evidence of substantial differential effects that may skew the representativeness of public health data along racial or ethnic lines compared to manual CICT.

## Discussion

Access to complete and trustworthy public health data is necessary for pandemic response. While previous work has focused on the relative benefits of manual CICT and digital contact tracing apps, we evaluate a third approach based on automated surveys. Our findings have substantial implications for pandemic response from an operational and health equity perspective.

First, automated CICT is less effective at collecting public health-relevant information. Despite the fact that both protocols attempted to elicit nearly identical information, surveys yielded substantially less information per-case about disease transmission (*e.g.*, travel), symptoms, and employer information that could indicate non-compliance with public health orders. Missing such instances could pose potential harms that public health departments must balance as the COVID-19 pandemic continues, and in preparation for future infectious disease outbreaks. Missing these instances may reduce the ability of public health officials to understand important details of disease spread and to craft effective policy.

Second, despite this, our analysis shows that automated CICT still collects significant amounts of data and provides critical population-level trends. This is relevant as a growing body of work has focused on the relationship between data quality and health inequity during the COVID-19 pandemic.^5^^,^^7^^,^^8^ A reasonable concern, then, is that alternative forms of CICT could skew data in a way that obscures health inequalities. However, while automated CICT may reduce individual case-level data, our analysis suggests that community-level patterns remain unchanged. Automated surveys may hence serve as useful and cost-effective fallbacks without necessarily increasing selection bias along racial and ethnic lines.

Third, automated CICT is easier to deploy and much more cost-effective and scalable. The average contact tracing call is 12.5 min long, and the median wage for an SCC communicable disease investigator is $38 per hour. Thus, the marginal cost per call, only taking into account contact tracer wages, is $7.91. In comparison, the marginal cost of sending the automated survey is negligible, generally between $0.01 and $0.05.^30^ In addition, the up-front cost of developing a manual CICT operation is many times more costly than the initial costs involved in developing the automated survey. While a full cost-benefit analysis is out of this paper's scope, it is clear that automated surveys are orders of magnitude more cost-effective than manual CICT.

Considering this altogether, public health departments which are already stopping manual CICT may find automated CICT to be a justifiable alternative for monitoring the pandemic. Alternatively, automated CICT could be used as a “first screening” that frees up critical human resources for other services that are harder to automate. A combined model of automated CICT and resource linkage may be fruitful and has been implemented in SCC for the most vulnerable ZIP codes.

We note several limitations of this study. First, the surge of cases due to the Omicron variant led to low CICT coverage in manual CICT ZIP Codes. This reduced our ability to study outcomes of interest, like the effect of contact tracing on COVID-19 case rates, which we present according to our study protocol in Appendix I. Second, through our imputation method we are able to analyze differential non-response for race/ethnicity questions; however, since similar methods are not available for other fields we are unable to investigate differential non-response for them. In addition, while BIFSG and similar methodologies are commonly used for race imputation, we observed worse performance in predicting Black and AIAN participants' race relative to other categories. Our study also does not speak to existing bias in measuring disparities when collecting information through manual CICT. It may be that certain subgroups are equally apprehensive towards manual and automated CICT. Additionally, the lack of a differential effect does not necessarily imply no effect at all. This is especially true given the small sample sizes for AIAN and other racial/ethnic categories, limiting our ability to study these subgroups and this study's generalizability to other populations. Within SCC, health disparities for the county's larger Latinx community were of particular concern,^31^ leading to the survey being fielded both in English and Spanish, which may explain the absence of differential effects. Further research into informational gaps for subgroups elsewhere is needed.

Notwithstanding these limitations, the stepped wedge design allows us to rigorously assess the informational impact of CICT. To our knowledge, this study is the first randomized trial involving the contact tracing of COVID-19, providing insights into programs that have received substantial public investment. We document large per-case information loss as a result of transitioning from manual to automated CICT. Due to the costs of contact tracing and perceived limitations in the face of high rates of community spread,^10^^,^^32^ many jurisdictions are phasing out conventional contact tracing, and we show that automated surveys may provide a cost-effective, scalable, and equitable option for pandemic surveillance.

## Contributors

D.E.H., D.O., C.R., A.D. and S.L.R. designed research; D.O., C.R., A.D. and D.E.H. performed research; C.R., D.O. and D.E.H. analyzed data; and C.R., D.E.H., D.O., A.D. and S.L.R. wrote the paper. All authors had access to and verified all the data in the study. D.E.H. was responsible for the decision to submit the manuscript.

## Data sharing statement

Aggregate data, along with the study protocol, is available online (see https://github.com/reglab/cict-stepped-wedge-replication). Data is in the form of a ZIP Code-by-week panel, including all outcomes, allowing for substantial replication of the main results. Due to privacy concerns, individual-level or race/ethnicity data are not available.

## Declaration of interests

The authors declare no competing interest.

## Appendix A Supplementary data

Appendices A–J

Captions for Supplementary Material

Stepped Wedge CICT Protocol
