# Supplementary material for: Automated vs. manual case investigation and contact tracing for pandemic surveillance: Evidence from a stepped wedge cluster randomized trial
Source: eClinicalMedicine. 2022 Nov 12;55:101726. doi: 10.1016/j.eclinm.2022.101726 (PMC9652032; doi:10.1016/j.eclinm.2022.101726)
Supplement: Appendices A–J [file mmc1.docx]

**Supplementary materials**

Case investigation and contact tracing (CICT) is an important tool for communicable disease control, both to proactively interrupt chains of transmission and to collect information for situational awareness. This study focuses on the utility of manual (*i.e.,* call-based) vs. automated (*i.e.*, survey-based) CICT for pandemic surveillance. While automated CICT is more cost-effective, and thus more sustainable given the length and magnitude of the COVID-19 pandemic, there are concerns about its informational value and potential to obscure health inequalities.

Between December 15, 2021 and February 5, 2022, a stepped wedge cluster randomized trial was run in which ZIP Codes progressively transitioned from manual to automated CICT. Eleven individual-level data fields on demographics and disease dynamics were observed for non-response. We observe 106,522 positive cases across 29 ZIP Codes.

Our analysis shows that automated CICT reduced overall collected information, as well as the response rate for individual fields. However, we found no evidence of differences in information loss by race or ethnicity. In other words, population-level trends remained stable. As a result, we argue that despite the reduction in information collected, automated CICT can serve as a useful and cost-effective alternative to manual CICT.

The following supplementary materials provide more detailed information on the intervention, outcomes, race imputation techniques, subgroup analyses, and robustness checks to our analyses.

**A. Call-based and automated survey contact tracing script excerpts**

The following excerpts are taken from the two conditions and demonstrate how both collect information on the same outcomes.

|  |  | **Manual CICT**  **(Phone Call)** | **Automated CICT**  **(Online Survey)** |
| --- | --- | --- | --- |
| **Question** | Introduction | I am with the Santa Clara County Department of Public Health calling in regards to your COVID-19 test result. First let me say that everything you and I talk about is confidential.  I need to let you know your COVID-19 test result has come back positive. This means you do have coronavirus disease or COVID-19. We are calling everyone who has tested positive in the state to share information about how to keep themselves and their families safe and to collect information so that we can prevent further spread of the virus. I also want to check on how you are doing, see if you need any support right now, and answer any COVID-19 questions you may have. | Hi, this is your Santa Clara County Department of Public Health reaching out about an important health issue.  We are reaching out to you because you have tested positive for COVID-19. This virus is very contagious, so it is important to keep it from spreading to others. The Santa Clara County Department of Public Health is working hard to slow the spread of COVID-19. You can help us by answering a few very important questions. The answers you give will help us protect you, the people in your house, and your community. |
|  | Symptoms | How are you feeling? Are you currently experiencing any symptoms? Some of the symptoms of COVID-19 are well known, while others are a little hard to recognize. So I’d like to read through a list of symptoms — could you tell me if you have experienced any of these symptoms? | Have you experienced any of these COVID-related symptoms recently? Click the arrow to view a list of COVID-related symptoms. At the end of the survey, we will provide you with information about COVID-19 symptoms and resources. |
|  | Travel History | Have you been outside of the United States in the 7 days before you tested positive or started having symptoms? If yes, which countries have you traveled to? | Have you been outside of the United States in the 2 weeks before you tested positive or started having symptoms? If yes, please select the countries you have traveled to. |
|  | Gathering History | In the 7 days before your symptoms started, did you do anything or go anywhere where you were around 4 or more people not living in your household? | In the 7 days before your symptoms started / or receiving your positive test, did you attend any large gatherings? |
|  | Ability to Isolate | What concerns you about being able to self-isolate? How sure are you that you are able to safely isolate at home? How safe do you feel in your home? | Are you able to safely self-isolate at home, away from others? This information can help us understand your current status at home and we may be able to help you. |
|  | Employer | What kind of work do you do? What kind of business or industry setting do you work in?^^[[1]](#footnote-0)^^ | For each of the jobs or workplaces where you were on-site (physically at the location and away from home) while you were contagious, please provide the following information.  We may need to contact your employer to let them know that you cannot work on-site due to a communicable disease. This section is optional. |
|  | Contacts Generated | Tell me what family members and friends you have seen. Who lives in your household? Is there anyone else who has been consistently present in your home during this time when you may have been most contagious? Who from your family or friends has visited you at home or other places? Was there anyone else you were around (within 6 feet) | Think about everyone who you have been close to since you became contagious. For example, think about people in your household, people you hugged or shared meals with, or anyone who was within 6 feet of you for 15 minutes or more over a 24-hour period. These are sometimes called "close contacts."  Have you had any close contacts since you became contagious? |
|  | Congregate Setting | Do you work or live in a place where many people from different families live, such as school-affiliated housing or residence hall, an assisted living or senior facility, group home, correctional facility, shelter, or something similar? | Since {contagiousDate}, did you work or live in a congregate setting? A "congregate setting" is any place where many people share a living or working space, like a nursing home, homeless shelter, or a jail or prison. |
|  | Race/ Ethnicity | How do you choose to identify your race and ethnicity? | Please share the following important information about yourself. This information helps us learn how COVID-19 is affecting your community.^^[[2]](#footnote-1)^^ |
|  | Language | Not specifically asked but recorded | Same as above |
|  | Sexual Orientation | How do you choose to identify your current sexual orientation or sexual identity? | Same as above |
|  | Gender | How do you choose to identify your current gender or gender identity? | Same as above |

*Table 4. Qualitative comparison of call-based and automated survey scripts for CICT. CICT = Case investigation and contact tracing;*

**B. Information loss time series breakdown**

Figure 3 depicts the change in data completeness overall, and for individual fields. These fields are:

1. Race/ethnicity
2. Employer name
3. Gender
4. Sexual orientation
5. Language
6. Signs/symptoms
7. Travel history
8. Gathering history
9. Ability to isolate
10. Whether they have any contacts
11. Whether they work or reside in a congregate setting (*e.g.*, nursing home, homeless shelter, jail, *etc.*)

*
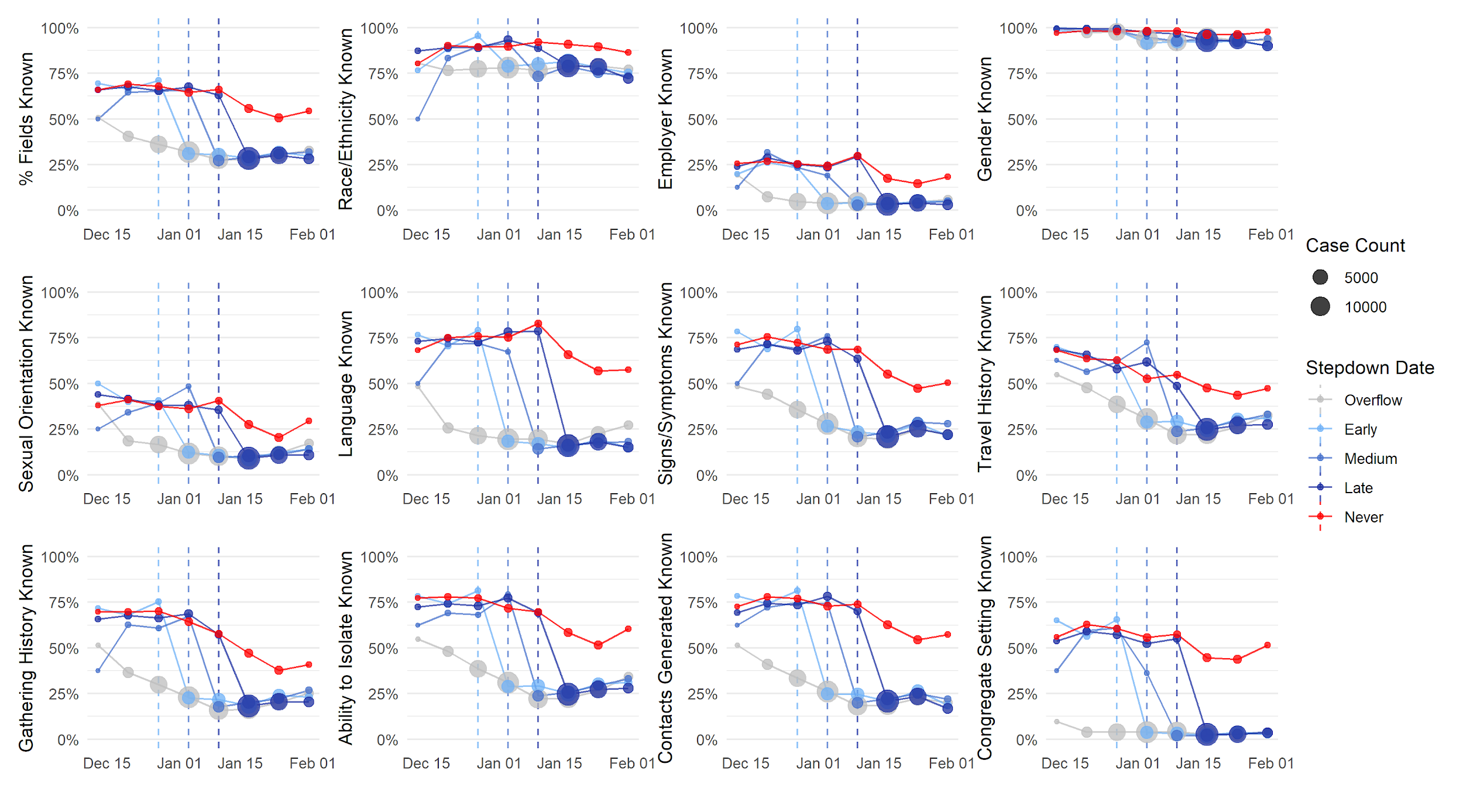
Figure 3. Step down group-by-week panel of response rate over time for all fields included in the overall response rate. Time series are colored according to the date that cluster of ZIP Codes randomly transitioned from manual to automated CICT, corresponding with the vertical dashed lines. The exception, which we include for reference, is the “Overflow” time series, which represents all cases randomized out of manual CICT due to capacity constraints. Point size corresponds to the number of cases for that cluster on that week. CICT = Case investigation and contact tracing.*

In alignment with our randomization protocol and formal analysis, the following figure shows the same set of time series segmented by Social Vulnerability Index (SVI) strata.


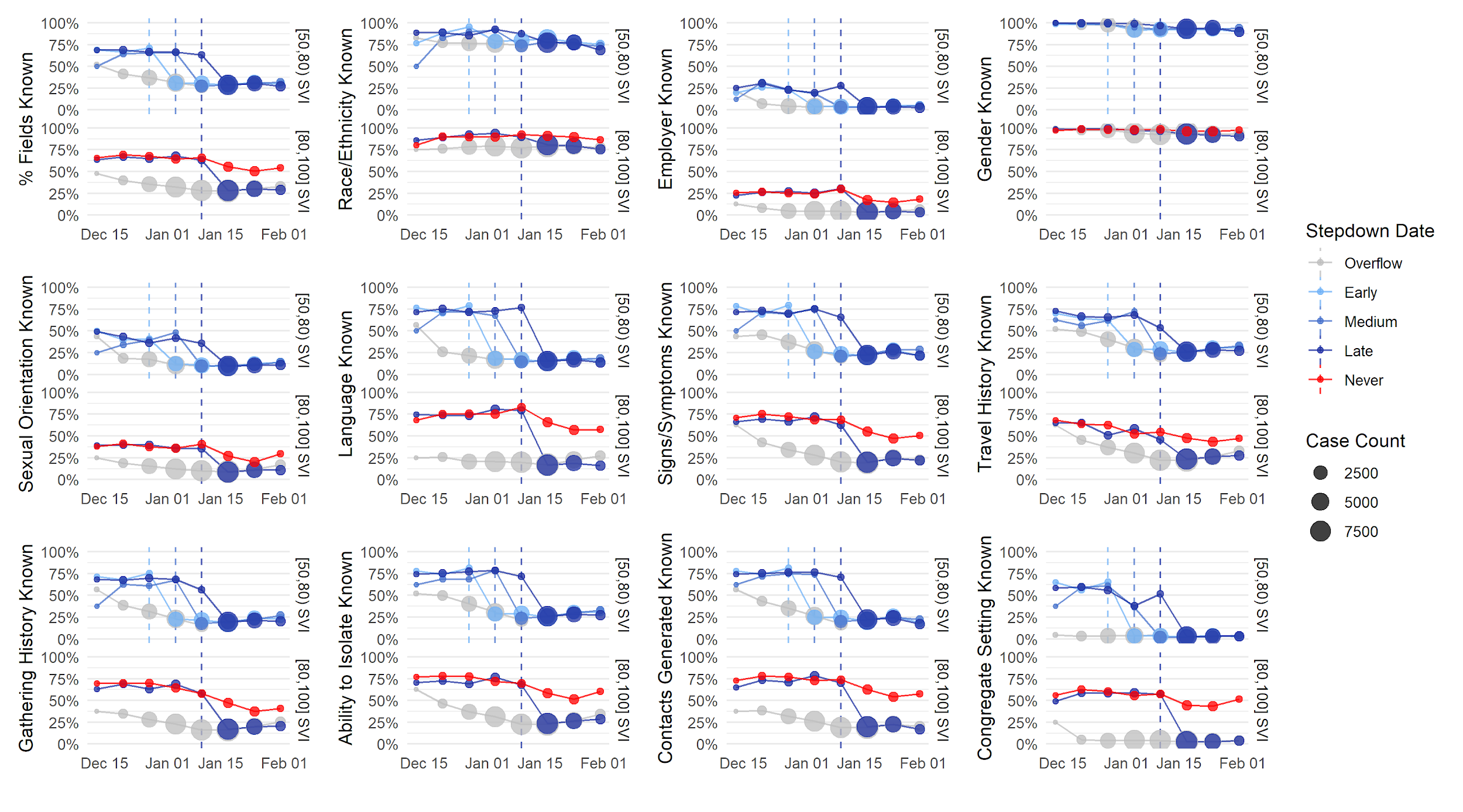


*Figure 4. Step down cluster-by-week panel of response rate over time for all fields included in the overall response rate, segmented by SVI strata. Lines are colored according to the date that cluster of ZIP Codes randomly transitioned from manual to automated CICT, corresponding with the vertical dashed lines. Point size corresponds to the number of cases for that cluster on that week. SVI = Social Vulnerability Index; CICT = Case investigation and contact tracing.*

**C. Manual and automated CICT coverage during the study period**


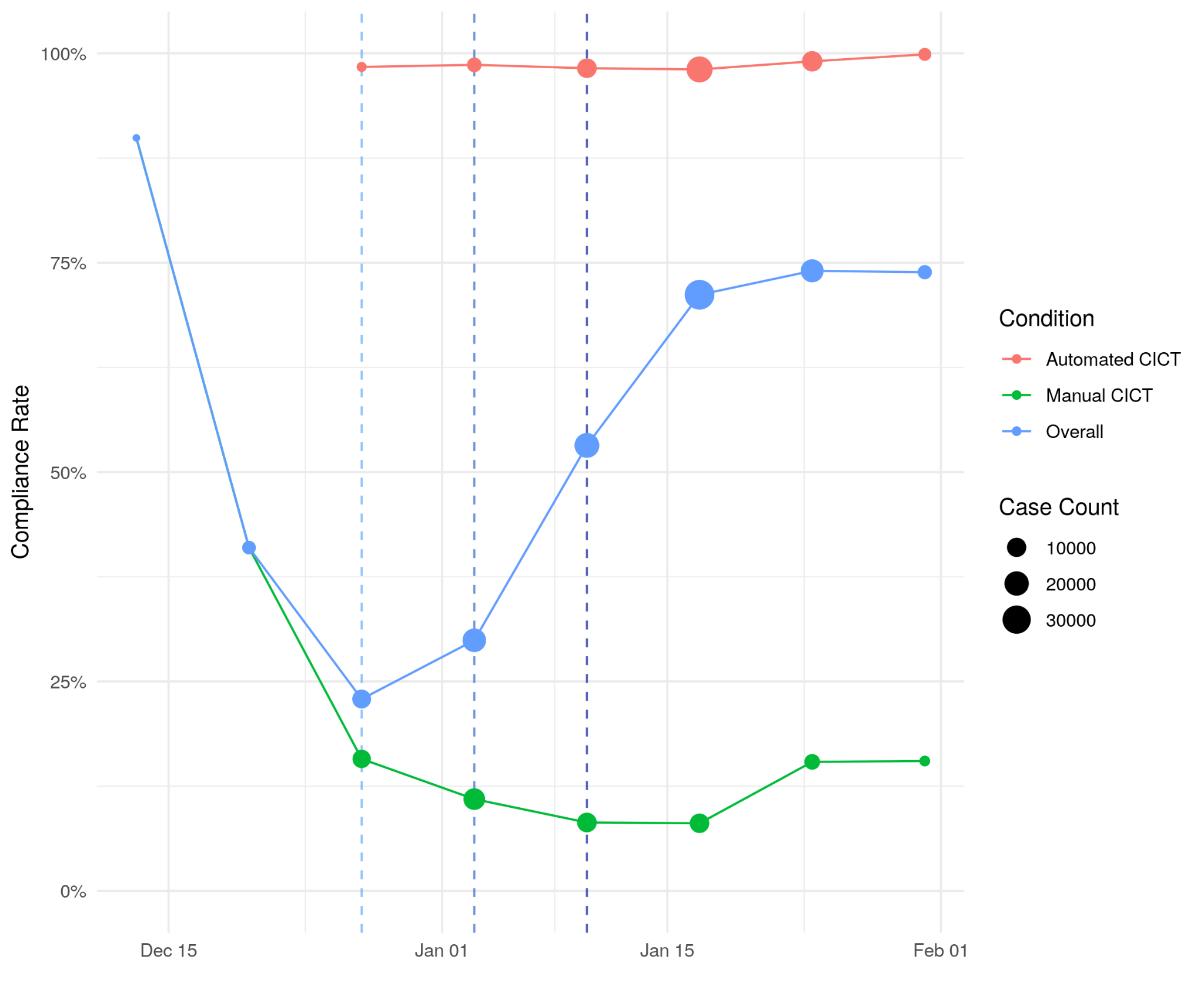


*Figure 5. Time series showing compliance rate (defined as the proportion of cases assigned to the correct condition) over time. To be assigned to manual CICT means that an attempt to call the participant was made. To be assigned to automated CICT means that the automated survey was sent. The red time series represents participants in the treatment condition (automated CICT); the green time series represents the control condition (manual CICT) and the blue time series represents the overall compliance rate. CICT = Case investigation and contact tracing.*

**D. Bayesian Improved First Name Surname Geocoding**

We use the standards on race and ethnicity set by the U.S. Office of Management and Budget (OMB) and Bayesian Improved First Name Surname Geocoding (BIFSG) to construct our race/ethnicity measure [(1)](https://www.zotero.org/google-docs/?4T8dkQ). BIFSG uses administrative data to calculate the probability of belonging to one of these racial/ethnic categories based on an individual’s first name, surname, and census block group (CBG). CBG is known for 83% of observations. When CBG isn’t known, BISFG falls back on first and surname information. We impute race/ethnicity as the most probable category based on BIFSG.

To evaluate BIFSG we first transform the observed race/ethnicity in CalCONNECT to match the OMB standards. When race/ethnicity is known, BIFSG correctly imputes the case’s race 68% of the time. We also note that BIFSG performs substantially worse for Black (n = 1071, 34% accuracy) and American Indian and Alaska Native (AIAN, n = 192 , 0% accuracy) individuals, though the small sample size of AIAN individuals likely contributes to the latter.

Figure 5 assesses the calibration of the estimated race probabilities. It compares the true probability that a case identifies as a particular race/ethnicity with the BIFSG predicted probability that a case does so. For example, of the individuals assessed to have a 50% probability of being Hispanic/Latino, roughly half of them self-report as such. Of course, this can only be done for individuals where race/ethnicity is known. As can be seen, there is a strong relationship between the predicted probability and empirical probability, though we observe worse calibration for White and Black individuals. AAPI and Hispanic/Latino calibration tends much closer to the 45 degree line. While the reliability of these calibrations is dependent on the extent to which racial and ethnic data is missing at random, BIFSG appears to perform reasonably for our analyses.

**
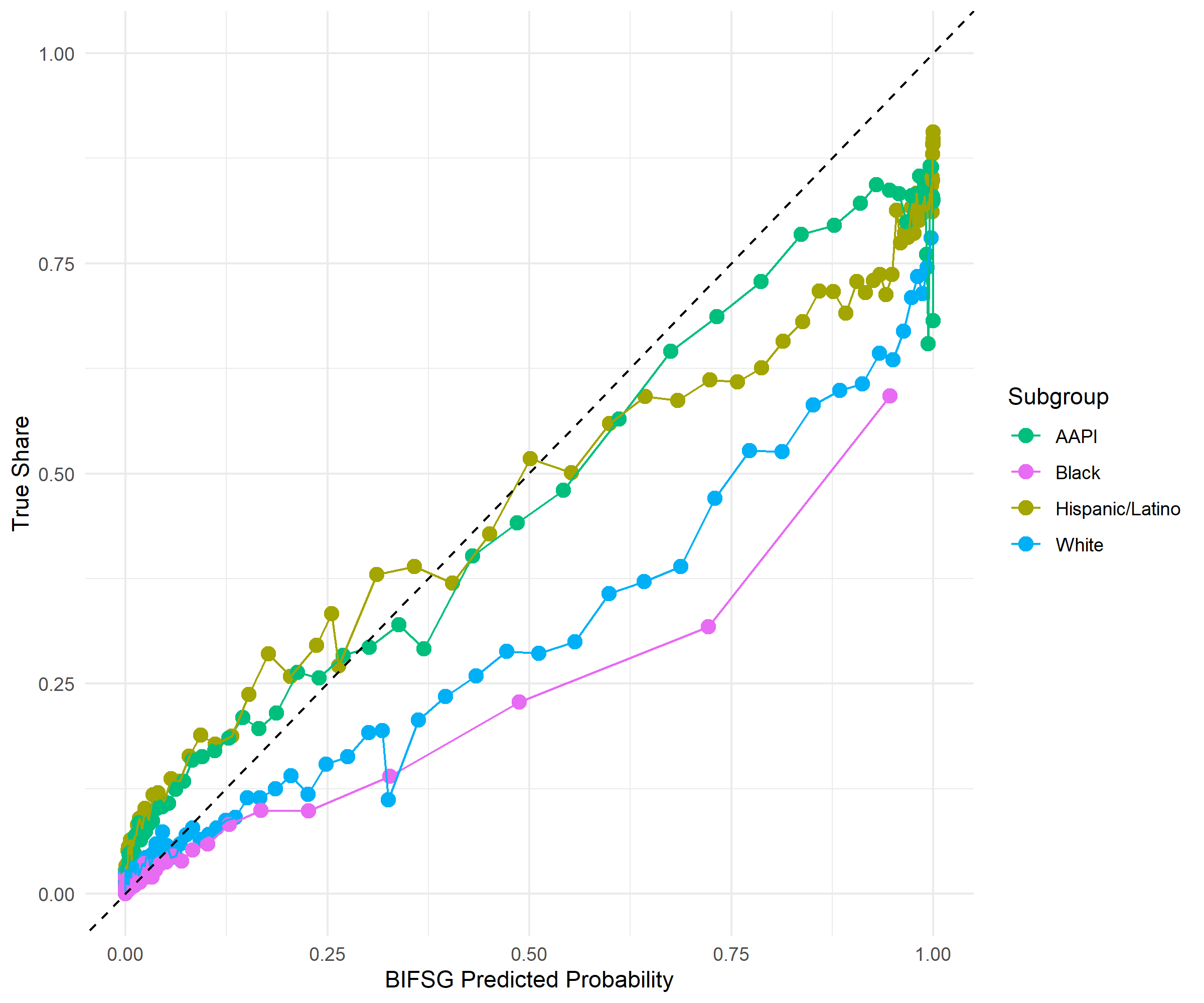
**

*Figure 6. Calibration of BIFSG imputations for cases with known race or ethnicity data in study period. For each racial/ethnic category, all observations are split into groups based on their predicted probability of belonging to that category (discretized into percentiles). This is shown on the x-axis. The y-axis represents the actual proportion of cases belonging to that category. A perfectly calibrated predictor would fall exactly on the dashed 45-degree line. BIFSG = Bayesian Improved First Name Surname Geocoding; AAPI = Asian American and Pacific Islander.*

**E. Conditional effect of contact tracing on information loss by race/ethnicity**

After imputing race/ethnicity with BIFSG, we then compute the conditional effect of contact tracing on information loss by BIFSG prediction. We only include BIFSG categories with sufficient sample sizes, excluding the categories AIAN and Other Race. While some estimates have large confidence intervals, we test whether *all* estimates are equal via a one-way ANOVA. Thus, a differential effect for a single race/ethnicity category is sufficient to reject the null hypothesis.

**
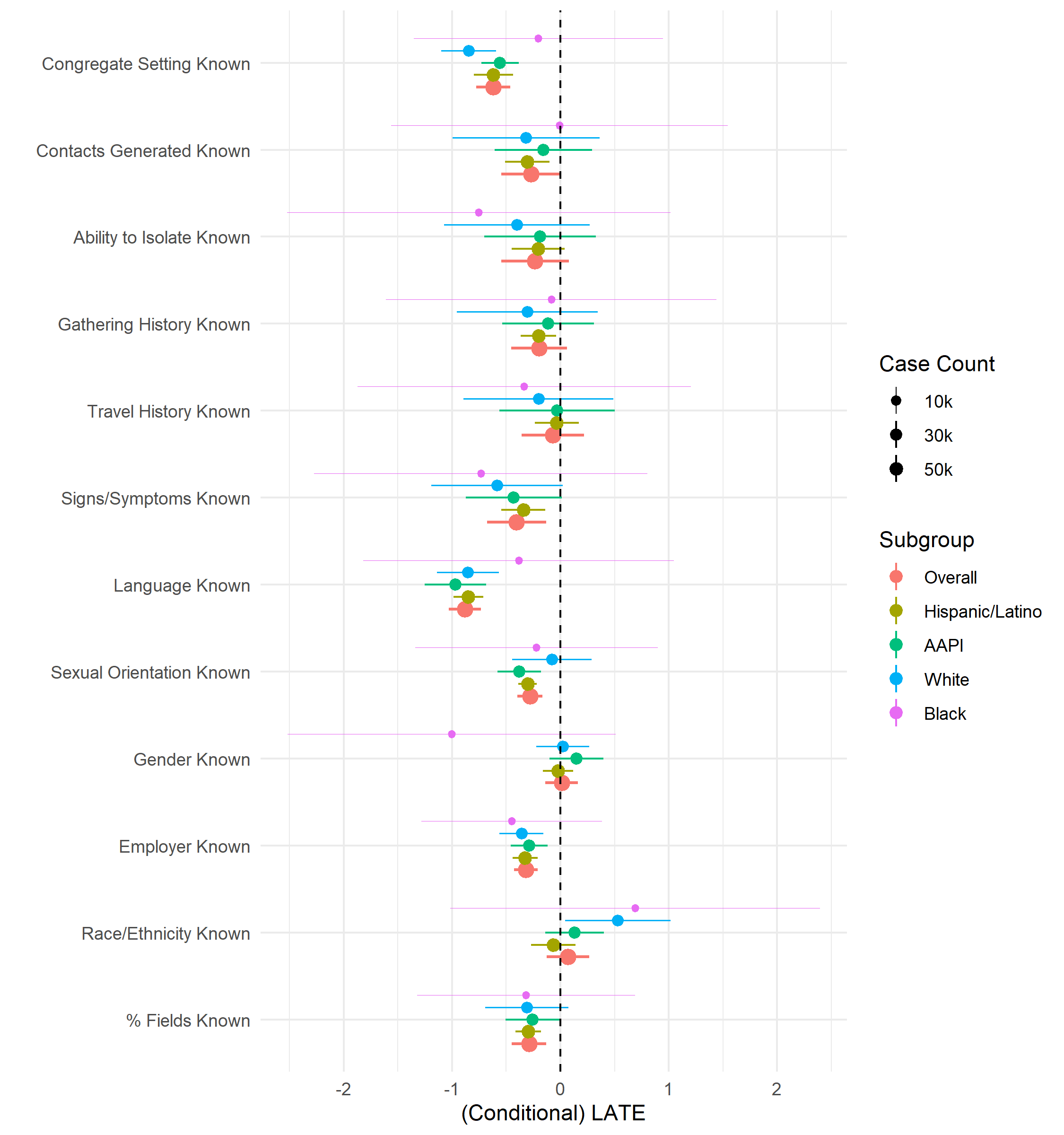
**

*Figure 7. LATE estimates of contact tracing on data completeness and conditional LATE (CLATE) estimates by predicted BIFSG race/ethnicity, with 95% confidence intervals. Sample is a repeated cross section at the individual-level using an estimator for difference-in-differences with multiple time periods, with standard errors clustered at the ZIP Code level. Due to insufficient sample sizes, CLATE estimates for the racial/ethnic categories “American Indian and Alaskan Native” and “Other Race” could not be computed. LATE = Local Average Treatment Effect; BIFSG = Bayesian Improved First Name Surname Geocoding; AAPI = Asian American and Pacific Islander.*

Due to the imprecision of BIFSG, Figure 7 shows these same analyses using self-reported race and only imputing race/ethnicity when it is not observed. Similarly, using a one-way ANOVA we are unable to reject the null hypothesis that conditional LATE estimates are equal for the overall completion rate (F(3,93170) = 1.36, *p* = 0.25). Thus, the absence of a significant finding is unlikely to be due to attenuation bias introduced by noise in BIFSG.


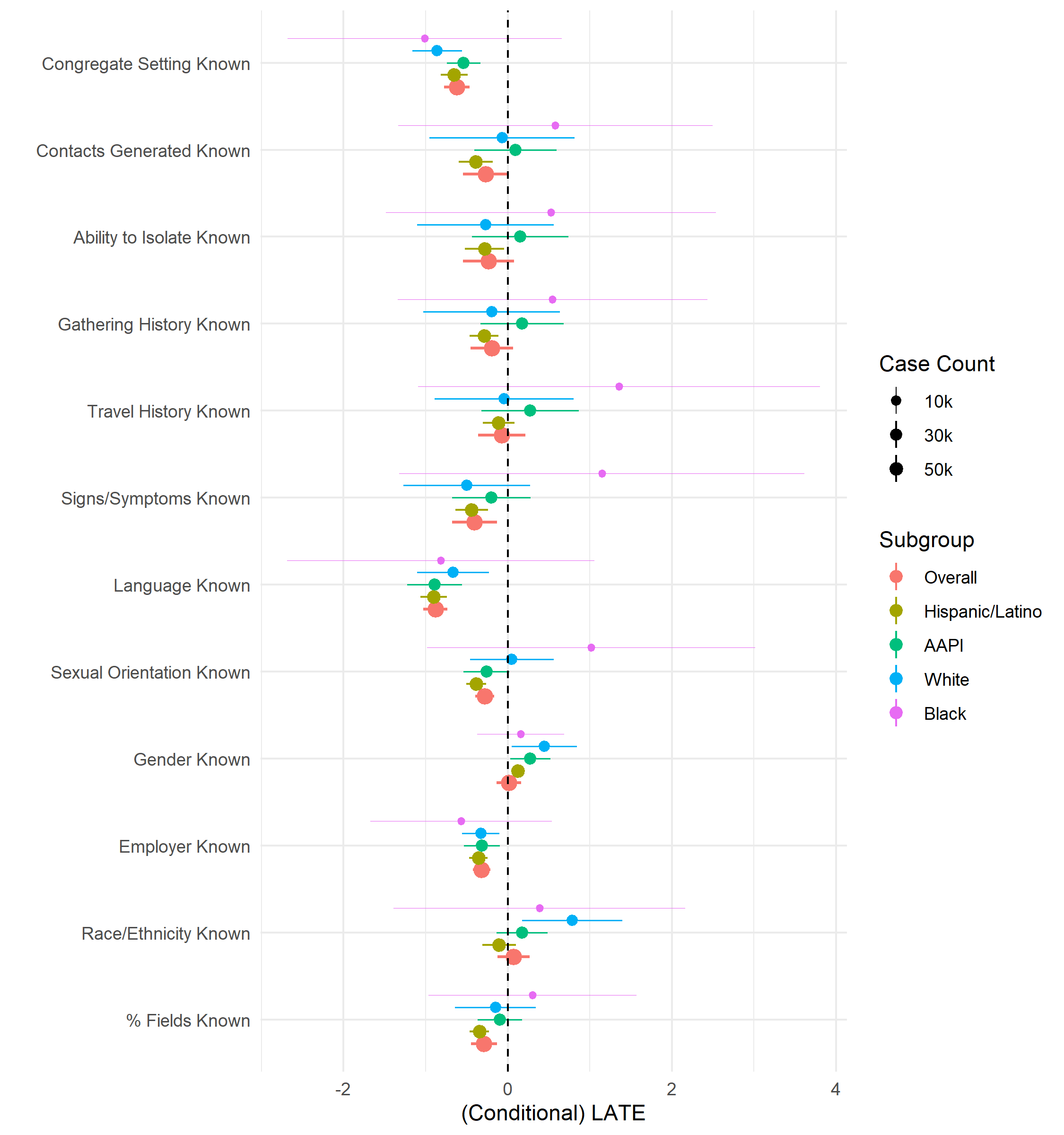


*Figure 8. LATE estimates of contact tracing on data completeness and conditional LATE (CLATE) estimates by observed race, only using BIFSG predictions when it is not observed, with 95% confidence intervals. Sample is a repeated cross section at the individual-level using an estimator for difference-in-differences with multiple time periods, with standard errors clustered at the ZIP Code level. Due to insufficient sample sizes, CLATE estimates for the racial/ethnic categories “American Indian and Alaskan Native” and “Other Race” could not be computed. LATE = Local Average Treatment Effect; BIFSG = Bayesian Improved First Name Surname Geocoding; AAPI = Asian American and Pacific Islander.*

As a final robustness check, we estimate the conditional LATE using a weighted regression approach. Instead of segmenting the data by subgroup and performing the IV analysis, we weight observations by the probability of belonging to the subgroup of interest. Thus, all observations in the data are included, but individuals who are unlikely to be a member of a racial or ethnic category have less influence on the estimate. These results are shown in Figure 8. As with our other subgroup analyses, we find no evidence for differential effects in the overall completion rate (F(3,426084) = 0.12, *p* = 0.95). Given the precision of these estimates and the large sample size, it is less likely that previous null results are due to a lack of statistical power.


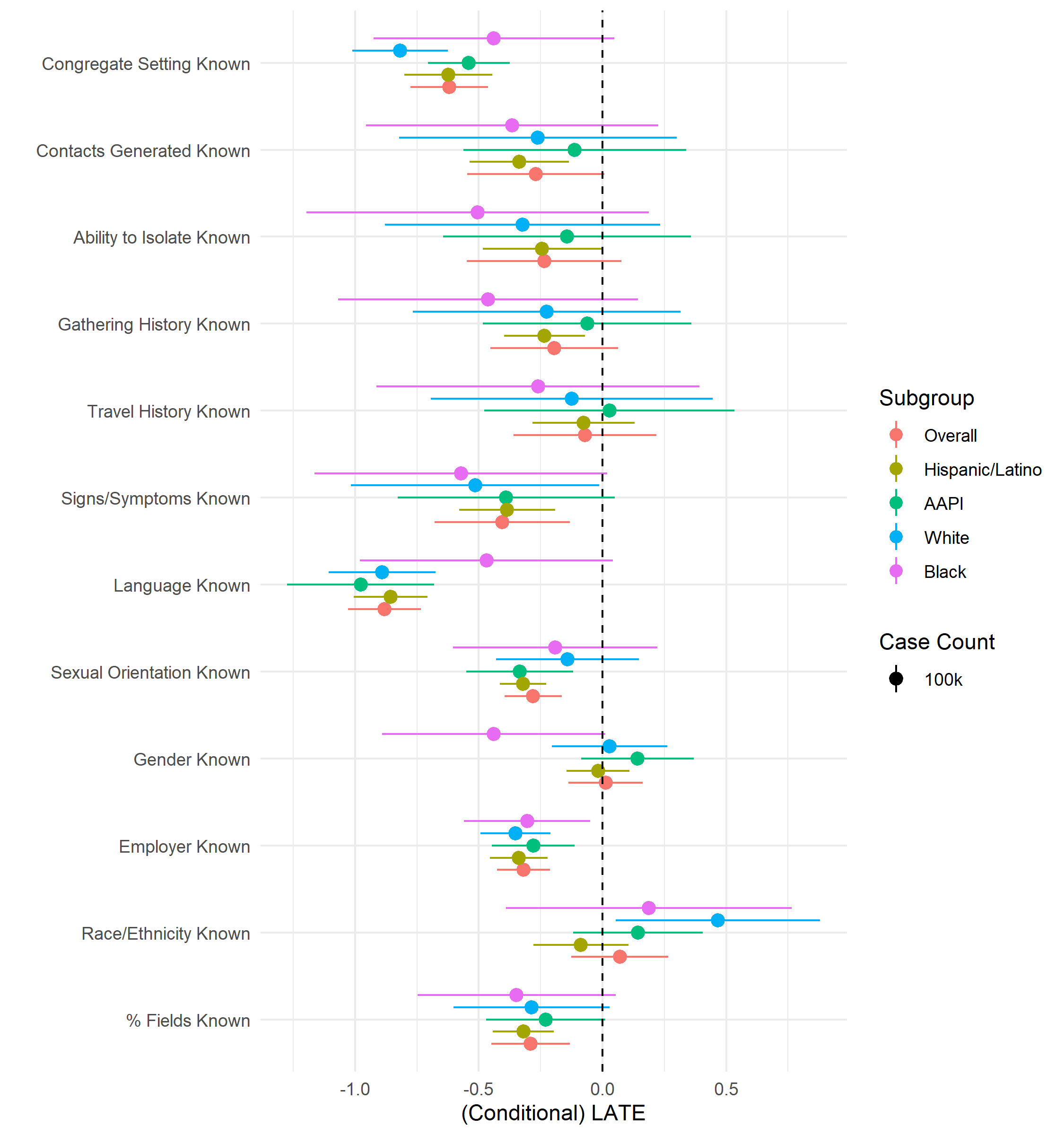


*Figure 9. LATE estimates of contact tracing on data completeness and conditional LATE (CLATE) estimates weighted by BIFSG race/ethnicity probabilities, with 95% confidence intervals. Sample is a repeated cross section at the individual-level using an estimator for difference-in-differences with multiple time periods, with standard errors clustered at the ZIP Code level. Due to insufficient sample sizes, CLATE estimates for the racial/ethnic categories “American Indian and Alaskan Native” and “Other Race” could not be computed. LATE = Local Average Treatment Effect; BIFSG = Bayesian Improved First Name Surname Geocoding; AAPI = Asian American and Pacific Islander.*

**F. Data completeness by treatment completed**

Our analyses randomize the intention to treat (ITT) positive cases with call-based or automated forms of contact tracing, and then scale the effect size to estimate the local average treatment effect (LATE) to account for non-compliance in the treatment assigned.

To further demonstrate that the information loss observed in our study is a result of the switch from call-based to automated contact tracing, Figure 9 visualizes the observational level of response by treatment *completed.* This differs from the preceding analyses, as being assigned to contact tracing, or receiving the automated survey, does not necessarily indicate completing those forms of contact tracing. For example, the case may simply not pick up the phone when called – in which case they did not complete, but were still assigned to contact tracing. Such non-adherence is likely confounded with other relevant factors. Figure 9 also shows that for some fields, like race/ethnicity or gender, the baseline rate of data completeness is quite high. This is due to individuals filling out a short questionnaire before getting their COVID-19 test, which asks these questions.

As Figure 9 shows, data completeness is generally highest when an individual completes call-based contact tracing. A notable exception to this is whether a case’s travel history is known, where data completeness is highest among those who submitted the automated survey. This is possibly because this question was asked at the beginning of the automated survey, and asked near the end of the call-based contact tracing (if there was time for it). However, in our analyses we still find a statistically significant reduction in data completeness for this field. This could be due to the fact that those in the call-based contact tracing protocol received a call and, in the event that they did not answer the call, multiple prompts to fill out the automated survey. Generally, these observational data indicate that the drop in data completeness is due to effectiveness of each protocol at collecting data.

*
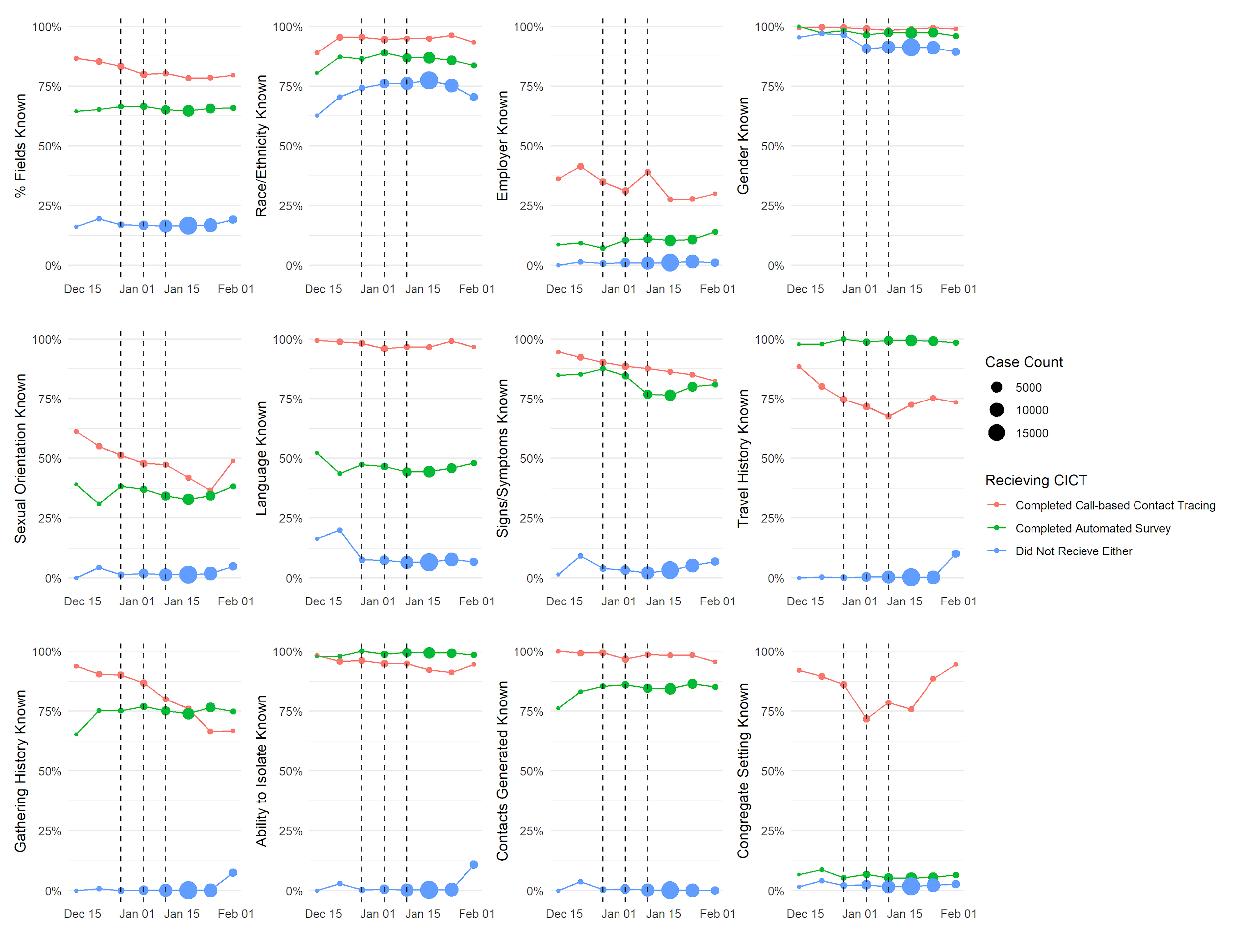
Figure 10. Treatment completed-by-week panel of response rate over time for all fields included in the overall response rate. Lines are colored according to the completed treatment.The vertical dashed lines correspond to the three step down dates. Point size corresponds to the number of cases who completed that treatment on that week. CICT = Case investigation and contact tracing.*

**G. Sensitivity analyses for instrumental variable estimates**

Here we test the robustness of our findings to various model specifications, using our instrumental variables (IV) approach to the two way fixed effects (TWFE) linear regression as a baseline. Controls include BIFSG probabilities, BIFSG imputed race, language, gender, and age. As can be seen, these controls do not dramatically alter our estimate of the coefficient of interest, due to the random nature of the CICT step down.

|  | Dependent variable: | | | | | |
| --- | --- | --- | --- | --- | --- | --- |
|  | % Fields Known | | | | | |
|  | No  Controls | BIFSG Probability Controls | Imputed Race Control | Spanish Speakers | Gender Control | Age  Control |
|  | (1) | (2) | (3) | (4) | (5) | (6) |
| Assigned to Automated | -0.29*** | -0.29*** | -0.29*** | -0.11** | -0.31*** | -0.28*** |
|  | (0.081) | (0.081) | (0.081) | (0.04) | (0.08) | (0.08) |
| High SVI | -0.003*** | -0.003*** | -0.003*** | 0.00 | 0.00 | -0.01*** |
|  | (0.00) | (0.00) | (0.00) | (0.00) | (0.00) | (0.00) |
| Observations | 106,522 | 106,522 | 106,522 | 5,126 | 99,898 | 106,446 |
| R^2^ | 0.128 | 0.128 | 0.128 | 0.146 | 0.135 | 0.182 |
| Adjusted R^2^ | 0.128 | 0.128 | 0.128 | 0.14 | 0.135 | 0.182 |
| Residual Std. Error | 0.243  (DF = 106,485) | 0.243  (DF = 106,480) | 0.243  (DF = 106,480) | 0.229  (DF = 5,089) | 0.242  (DF = 99,858) | 0.235  (DF = 106,408) |
|  | *p<0.1; **p<0.05; ***p<0.01 | | | | | |

*Table 5. The robustness of automated CICT's effect on information loss including various control variables. Outcome variable is the percentage of fields filled out. Regression (1) presents the standard IV results; (2) includes the BIFSG probabilities for each racial/ethnic category; (3) includes the most probable race according to BIFSG as a control; (4) represents the effect for known Spanish speakers; (5) includes gender (when known) as a control; (6) includes age (when known) as a control. All balance variables are transformed according to Section 3. Effects are estimated via a two way fixed effects linear regression, and control for SVI Strata. ZIP Code randomization is used as an instrument for the treatment which is ultimately assigned. Standard errors are clustered at the ZIP Code level in accordance with our randomization protocol and are presented in parentheses below estimates. BIFSG = Bayesian Improved First Name Surname Geocoding; SVI = Social Vulnerability Index; DF = Degrees of freedom; CICT = Case investigation and contact tracing; IV = Instrumental variables.*

**H. Per-protocol analysis**


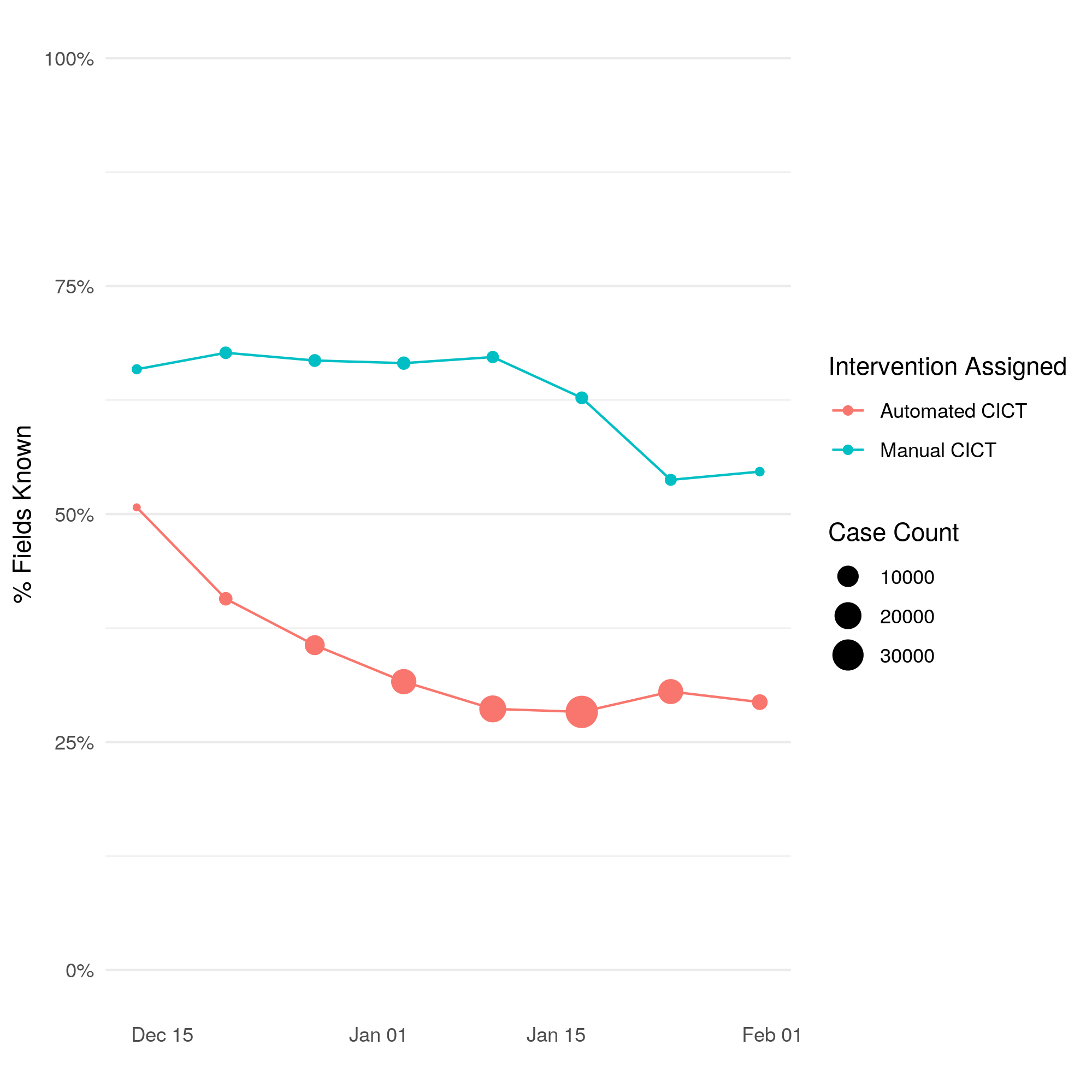


*Figure 11. Treatment-by-week panel of response rate over time for all fields included in the overall response rate. Lines are colored according to the completed treatment.The vertical dashed lines correspond to the three step down dates. Point size corresponds to the number of cases who completed that treatment on that week. CICT = Case investigation and contact tracing.*

In addition to our ITT and IV estimates, we also estimate the as-treated “effect” of automated CICT on information loss. In the context of randomized control trials, instrumental variable approaches are generally preferred for handling non-compliance. This is because it is possible, however unlikely, that contact tracers selectively assigned patients to manual CICT if they thought they would provide information. This confounding would bias our results. However, when comparing individuals on the basis of the treatment which they received, we find similar results. A per protocol analysis shows a 32pp reduction in completion rate.

|  | Dependent variable: |
| --- | --- |
|  | % Fields Known |
|  | As-Assigned Estimate |
| Assigned to Automated | -0.323*** |
|  | (0.01) |
| High SVI | -0.004*** |
|  | (0.00) |
| Observations | 106,522 |
| R^2^ | 0.129 |
| Adjusted R^2^ | 0.129 |
| Residual Std. Error | 0.243 (DF = 106,485) |
| F Statistic | 438.574*** (DF = 36; 106,485) |
|  | *p<0.1; **p<0.05; ***p<0.01 |

*Table 6. Two way fixed effects linear regression comparing those as-treated to manual and automated CICT. Standard errors are clustered at the ZIP Code level in accordance with our randomization protocol and are presented in parentheses below estimates. SVI = Social Vulnerability Index; CICT = Case investigation and contact tracing.***I. Community transmission**

As previously noted, we were not well powered to detect changes in ZIP Code COVID-19 case rates in this study. However, as per our study protocol, we present preliminary results investigating this. As can be seen visually, there is no discernible change in COVID-19 case rates (per 100k) in the periods following a cluster’s transition from manual to automated CICT. There is no statistically significant effect when analyzing the data via a two way fixed effects regression.


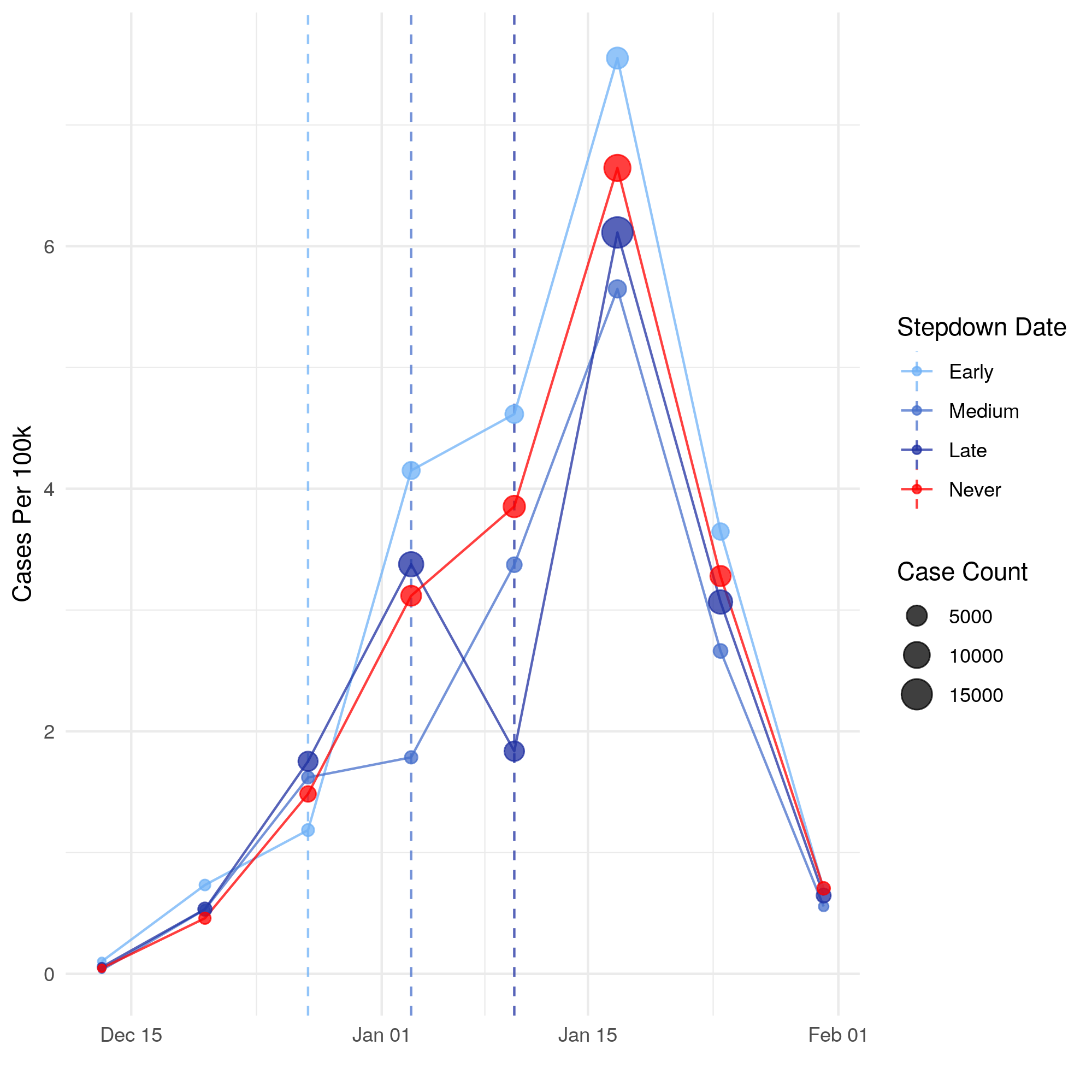


*Figure 12. Step down cluster-by-week panel of COVID-19 cases per 100k over time. Lines are colored according to the date that cluster of ZIP Codes randomly transitioned from manual to automated CICT, corresponding with the vertical dashed lines. Point size corresponds to the total number of cases for that cluster on that week. CICT = Case investigation and contact tracing.*

**J. SVI strata analysis**

Given that our randomization is stratified by Social Vulnerability Index (SVI), here we analyze the stepped wedge trial by SVI strata. This replicates two traditional difference-in-differences research designs, where clusters progressively transition from the control to the treatment condition, leaving one “never treated” cluster. To retain a “never treated” cluster in the [50,80) SVI cohort we filter out the weeks after the last cluster steps down. This setup can be seen in Figure 13 below. As can be seen, in both instances we observe similar effect sizes.


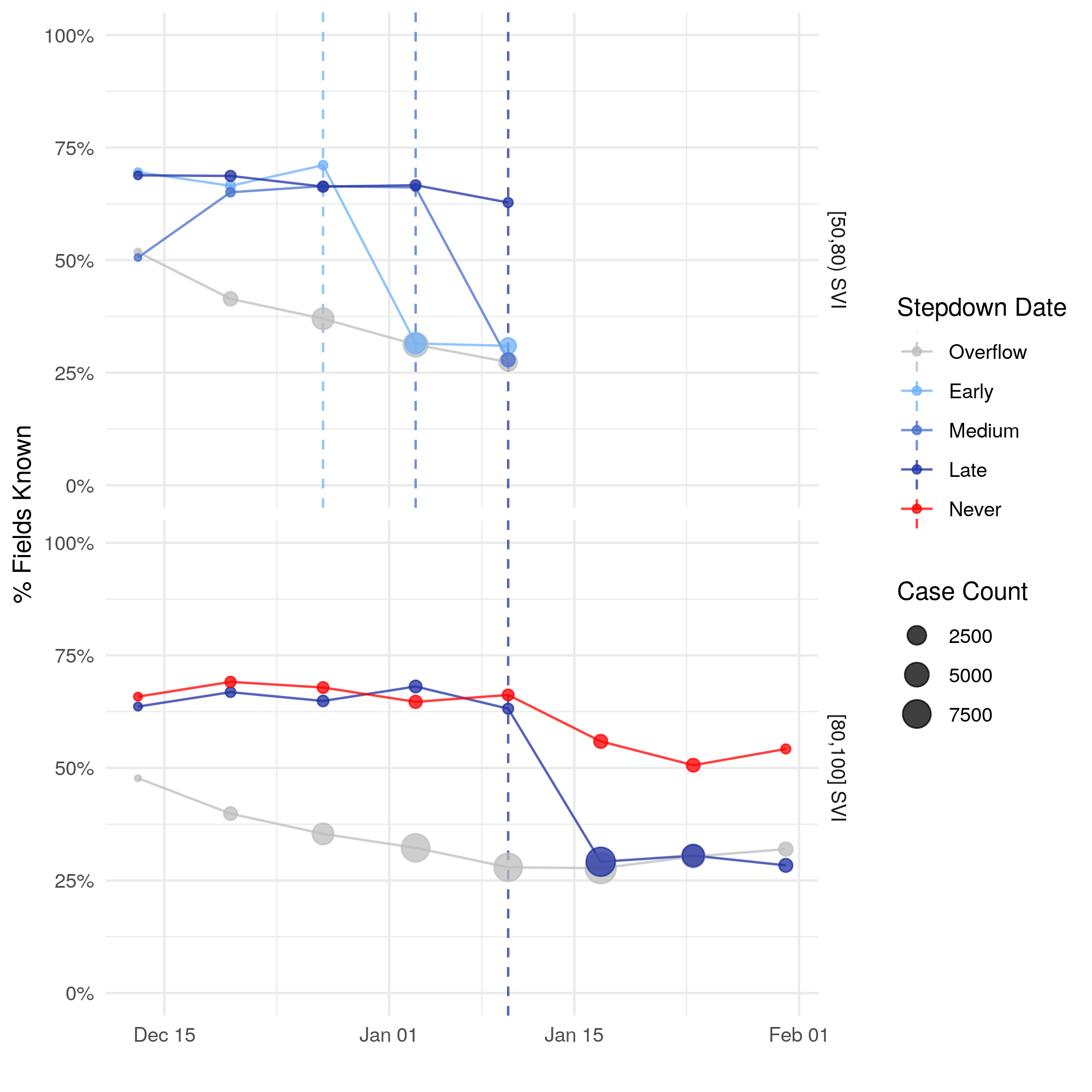


*Figure 13. Step down cluster-by-week panel of response rate over time for all fields included in the overall response rate, segmented by SVI strata. Observations are removed after the last cluster in the [50,80) SVI stratum step down. Lines are colored according to the date that cluster of ZIP Codes randomly transitioned from manual to automated CICT, corresponding with the vertical dashed lines. Point size corresponds to the number of cases for that cluster on that week. SVI = Social Vulnerability Index; CICT = Case investigation and contact tracing.*

|  | Dependent variable: | | | |
| --- | --- | --- | --- | --- |
|  | Percent Fields Known | | | |
|  | [50,80) SVI ITT | [50,80) SVI IV | [80,100] SVI ITT | [80,100] SVI IV |
|  | (1) | (2) | (3) | (4) |
| Randomized to Automated | -0.037** |  | -0.024*** |  |
|  | (0.01) |  | (0.01) |  |
| Assigned to Automated |  | -0.765*** |  | -0.235*** |
|  |  | (0.27) |  | (0.08) |
| Observations | 20,763 | 20,763 | 54,632 | 54,632 |
| R^2^ | 0.062 | -0.029 | 0.035 | 0.145 |
| Adjusted R^2^ | 0.061 | -0.03 | 0.035 | 0.145 |
| Residual Std. Error | 0.269  (DF = 20,741) | 0.281  (DF = 20,741) | 0.257  (DF = 54,612) | 0.242  (DF = 54,612) |
| F Statistic | 64.923***  (DF = 21; 20,741) |  | 104.900***  (DF = 19; 54,612) |  |
|  | *p<0.1; **p<0.05; ***p<0.01 | | | |

*Table 7. SVI Stratified estimates for the effect of automated CICT on per-case completeness spanning eleven critical data fields. Outcome variable is the percentage of fields filled out. Regression (1) presents the ITT (as randomized) effect, via two way fixed effects linear regression for the [50,80) SVI ZIP Codes. Regression (2) presents the LATE which also uses a two way fixed effects linear regression, but uses the ZIP Code randomization as an instrument for the treatment which is ultimately assigned. Regressions (3) and (4) repeat this but for the [80,100] SVI stratum. To replicate a traditional difference-in-differences design we remove observations after the last cluster in the [50,80) SVI stratum step down. Standard errors are presented in parentheses below estimates. SVI = Social Vulnerability Index; ITT = Intention to treat; IV = Instrumental variables; DF = Degrees of freedom; CICT = Case investigation and contact tracing; LATE = Local average treatment effect.*

**References**

[1. Budget O of M and. Standards for maintaining, collecting, and presenting federal data on race and ethnicity. Fed Regist. 1997;62:58781–90.](https://www.zotero.org/google-docs/?rVlxny)

1. This question is used to collect multiple fields regarding the case’s employer and occupation, including the name of the employer. [↑](#footnote-ref-0)
2. This question precedes numerous drop down menus and text inputs which allows the case to input their demographic attributes. [↑](#footnote-ref-1)
