# Supplementary material for: Automated vs. manual case investigation and contact tracing for pandemic surveillance: Evidence from a stepped wedge cluster randomized trial
Source: eClinicalMedicine. 2022 Nov 12;55:101726. doi: 10.1016/j.eclinm.2022.101726 (PMC9652032; doi:10.1016/j.eclinm.2022.101726)
Supplement: Captions for Supplementary Material [file mmc2.docx]

REVISED Supplemental Materials -> Supplementary Materials for Automated vs. Manual Case Investigation and Contact Tracing for Pandemic Surveillance: Evidence from a Stepped Wedge Cluster Randomized Trial

Stepped Wedge CICT Protocol -> Study Protocol for Automated vs. Manual Case Investigation and Contact Tracing for Pandemic Surveillance: Evidence from a Stepped Wedge Cluster Randomized Trial
