# Supplementary material for: Automated vs. manual case investigation and contact tracing for pandemic surveillance: Evidence from a stepped wedge cluster randomized trial
Source: eClinicalMedicine. 2022 Nov 12;55:101726. doi: 10.1016/j.eclinm.2022.101726 (PMC9652032; doi:10.1016/j.eclinm.2022.101726)
Supplement: Stepped Wedge CICT Protocol [file mmc3.pdf]

Protocol: Automated vs. Manual Case Investigation and Contact Tracing for Pandemic Surveillance: Evidence from a Stepped Wedge Cluster Randomized Trial

Principal Investigator:

Daniel E. Ho, JD, PhD[1]
Stanford University
Stanford, CA, USA

Email: dho@law.stanford.edu

Coauthors: Cameron Raymond[1]; Derek Ouyang[1]; Sarah L. Rudman[2]; Alexis D'Agostino[2]

[1] Stanford University
[2] Santa Clara County Public Health Department

Background:

Case investigation and contact tracing (CICT) is an important tool for communicable disease control, both to proactively interrupt chains of transmission and to collect information for situational awareness. To date in the COVID-19 pandemic, there have been no randomized control trials involving COVID-19 CICT. Rigorous evidence is needed to help public health departments balance cost, accuracy, and completeness of data derived from CICT, as it is traditionally extremely resource-intensive.

Aim:

To evaluate the effect of manual (*i.e.*, call-based) and automated (*i.e.,* survey-based) approaches to CICT as it relates to data collection needed for situational awareness.

An original aim of this study was to evaluate the effect of CICT on COVID-19 case rates. However, our power analysis indicated that we were not able to do so. As such, we focus on the informational value of CICT.

Study Design:

This study utilizes a stepped wedge (randomized rollout) cluster randomized design. This was due to Santa Clara County Public Health Department's prior decision to transition from manual to automated CICT. The design both facilitates an evaluation of CICT, while still ensuring that the most vulnerable ZIP codes receive any possible benefits of contact tracing for the longest duration.

The randomization occurs at the ZIP Code level. We first stratify the county by social vulnerability index (SVI) into medium SVI [50-80), and high SVI (80+) ZIP Codes. ZIP Codes with SVI [50,80) were randomized into early, medium, and late step down groups. ZIP Codes with SVI [80,100] were randomized into a late step down group and a never step down group. This was done by the researchers

with a random number generator prior to the study period. The study period is December 15, 2021 to February 5, 2022.

Intervention:

Positive cases in this study receive one of two interventions.

Manual CICT (call-based or control) involves a telephone call from a trained contact tracer who gathers information surrounding the case. Depending on capacity, contact tracers will attempt to call an individual up to six times. If a call is not answered after the first day, the automated survey is sent as well.

Automated CICT (survey-based or treatment) is sent via text message/email. Surveys are answered on a web browser and consist of free-text, multiple choice, and drop down inputs.

Outcomes:

Individual-level data is entered in the state-managed system used to record and investigate COVID-19 cases in California (CalCONNECT). We focus on eleven data fields deemed especially important for pandemic response. These are necessary to understand population-level trends in the pandemic, as well as disease dynamics:

1. Race/ethnicity
2. Employer name
3. Gender
4. Sexual orientation
5. Language
6. Signs/symptoms
7. Travel history
8. Gathering history
9. Ability to isolate
10. Whether they have any contacts
11. Whether they work or reside in a congregate setting (*e.g.*, nursing home, homeless shelter, jail, *etc.*)

Each field is observed for non-response, including “unknown”, “declined to answer”, and null. To reduce question-specific variance, our primary outcome is the percentage of fields completed.

Sample Size:

The study’s timeline was based on SCCs existing decision to transition from manual to automated CICT. Based on prior Monte Carlo simulations using historical data, given 100k cases, we have 80% power to detect 12 percentage point changes in information loss assuming manual CICT coverage of 10%.

Analysis Plan:

To formally assess the causal effect of automated CICT on information loss, we adopt a difference-in-differences approach. This compares the post-step down group to those in that same group pre-step down, as well as those in control ZIP codes. By taking into account temporal trends and differences in ZIP Code baseline rates, we can estimate the effect of CICT on an intent-to-treat (ITT) basis.

We estimate the ITT effect using a two way fixed effects (TWFE) linear regression with robust standard errors clustered at the ZIP Code level to account for our clustered treatment assignment and heteroscedasticity. In addition to week and ZIP Code fixed effects we also control for SVI stratum, taking into account our randomization protocol.

To model non-compliance in the treatment assigned (due to capacity constraints in manual CICT), we also estimate the local average treatment effect (LATE) adopting an instrumental variable (IV) approach. We use our randomization protocol as an instrument for the treatment assigned, using the common two-stage least squares (TSLS) estimator. As with our ITT analysis, we include week, ZIP Code, and SVI stratum fixed effects and cluster the standard errors at the ZIP Code level.
